# Situating the left-lateralized language network in the broader organization of multiple specialized large-scale distributed networks

**DOI:** 10.1152/jn.00753.2019

**Published:** 2020-09-23

**Authors:** Rodrigo M. Braga, Lauren M. DiNicola, Hannah C. Becker, Randy L. Buckner

**Affiliations:** ^1^Department of Psychology, Center for Brain Science, Harvard University, Cambridge, Massachusetts; ^2^Department of Neurology and Neurological Sciences, Stanford University, Stanford, California; ^3^The Computational, Cognitive, and Clinical Neuroimaging Laboratory, Hammersmith Hospital Campus, Imperial College London, London, United Kingdom; ^4^Athinoula A. Martinos Center for Biomedical Imaging, Massachusetts General Hospital, Charlestown, Massachusetts; ^5^Department of Radiology, Harvard Medical School, Boston, Massachusetts; ^6^Department of Psychiatry, Massachusetts General Hospital, Charlestown, Massachusetts

**Keywords:** Broca’s area, distributed association networks, intrinsic functional connectivity, language, Wernicke’s area

## Abstract

Using procedures optimized to explore network organization within the individual, the topography of a candidate language network was characterized and situated within the broader context of adjacent networks. The candidate network was first identified using functional connectivity and replicated across individuals, acquisition tasks, and analytical methods. In addition to classical language regions near the perisylvian cortex and temporal pole, regions were also observed in dorsal posterior cingulate, midcingulate, and anterior superior frontal and inferior temporal cortex. The candidate network was selectively activated when processing meaningful (as contrasted with nonword) sentences, whereas spatially adjacent networks showed minimal or even decreased activity. Results were replicated and triplicated across two prospectively acquired cohorts. Examined in relation to adjacent networks, the topography of the language network was found to parallel the motif of other association networks, including the transmodal association networks linked to theory of mind and episodic remembering (often collectively called the default network). The several networks contained juxtaposed regions in multiple association zones. Outside of these juxtaposed higher-order networks, we further noted a distinct frontotemporal network situated between language regions and a frontal orofacial motor region and a temporal auditory region. A possibility is that these functionally related sensorimotor regions might anchor specialization of neighboring association regions that develop into a language network. What is most striking is that the canonical language network appears to be just one of multiple similarly organized, differentially specialized distributed networks that populate the evolutionarily expanded zones of human association cortex.

**NEW & NOTEWORTHY** This research shows that a language network can be identified within individuals using functional connectivity. Organizational details reveal that the language network shares a common spatial motif with other association networks, including default and frontoparietal control networks. The language network is activated by language task demands, whereas closely juxtaposed networks are not, suggesting that similarly organized but differentially specialized distributed networks populate association cortex.

## INTRODUCTION

The association cortex comprises a mosaic of distributed networks that each interconnect regions in prefrontal, parietal, temporal, and midline cortices (Goldman-Rakic 1988; see also Mesulam 1981; 1990). The spatial motif of regions in each of these distributed cortical zones showing connectivity is replicated across neighboring cortical locations, leading to a parallel organization of networks (Braga and Buckner 2017; Margulies et al. 2016; Power et al. 2011; Yeo et al. 2011). One hypothesis is that the broad organization of higher-order association cortex is established early in development, with subsequent specialization of cortical zones into distinct networks by activity-dependent processes (Buckner and DiNicola 2019). Although much work has previously estimated the spatial relations between multiple higher-order association networks (e.g., see Margulies et al. 2016), open questions regard *1*) the extent to which the language network possesses features similar to other distributed association networks and *2*) how the language network fits within the spatial macroscale organization of the cerebral cortex.

The description of interconnected anterior and posterior language regions inspires much of the contemporary study of human brain networks. The classic perisylvian language system, initially identified through case studies of patients with aphasia, included an extended region encompassing inferior frontal gyrus (IFG) just rostral to motor cortex (i.e., Broca’s area) and the posterior superior temporal cortex (pSTC; i.e., Wernicke’s area; see Geschwind 1970). Quantitative lesion mapping (Bates et al. 2003; Mirman et al. 2015) and study of primary progressive aphasia (Mesulam et al. 2013) have highlighted the importance of rostral regions of temporal association cortex extending to the temporal pole (TP) and additional regions at and around traditionally defined Broca’s area. Taken together, classical and contemporary findings on the anatomy of language function support a specialized, left-lateralized network that involves multiple distributed anterior and posterior association regions.

Task activation studies of language based on group averages yield an estimate of regions involved in language function that is in many ways consistent with the clinical literature (e.g., see Blank et al. 2002; Ferstl et al. 2008; Hickok and Poeppel 2007; Petersen et al. 1988; Wise et al. 1991). Findings converge on a left-lateralized network active during speech reception and production, with regions distributed in anterior and posterior zones that include pSTC, often extending rostrally to the temporal pole, and prefrontal regions that prominently include the IFG. It is intriguing that this canonical set of language regions broadly adheres to the general motif of other association networks (Goldman-Rakic 1988; Margulies et al. 2016; Power et al. 2011; Yeo et al. 2011). Furthermore, the proximity of language regions in the IFG and temporal association cortex to orofacial motor and auditory cortices (Geschwind 1970; Krubitzer 2007) may have relevance to the development of language pathways (see also Hickok and Poeppel 2007). Building upon the foundations provided by these prior studies, a core goal of the present work is to use within-individual neuroimaging approaches to characterize the detailed spatial organization of the language network in relation to other nearby functionally distinct networks.

A further motivation for exploring the detailed organization of a language network is that group-based studies frequently reveal that the same (or nearby) IFG regions are activated by both linguistic and nonlinguistic task demands. This observation led to suggestions that certain parts of the estimated language system act as domain-flexible resources supporting controlled processing (e.g., see Burianova and Grady 2007; Gold and Buckner 2002; Hein and Knight 2008; Poldrack et al. 1999; and Thompson-Schill et al. 1997; see Blumstein and Amso 2013 for relevant discussion). One possibility is that distinct regions are blurred together in group-averaged data. Of critical importance is that when functional zones are defined within individuals, distinct language-specific regions can be defined within IFG that lay in close proximity to and are surrounded by less domain-specialized regions (Fedorenko et al. 2010, 2012) that are typically considered part of a separate system referred to as the “multiple-demand” or “frontoparietal control” network (FPN; see Duncan 2010 and Vincent et al. 2008). The implication is that individually focused analyses can resolve details of regional specialization, particularly in association regions like the IFG, that may have densely packed functional zones that vary in location across individuals (Fedorenko et al. 2012; Mueller et al. 2013; see also Jakobsen et al. 2018).

The close juxtaposition of multiple functionally distinct regions near language regions may also have complicated group-based functional connectivity estimates of network organization. Using data-driven algorithms that “parcellate” the cortex into discrete networks, group-averaged analyses indicate that the association cortices typically comprise around five major networks (Doucet et al. 2011; Power et al. 2011; Yeo et al. 2011), none of which is an unequivocal candidate for a language system (see Ji et al. 2019 for a discussion). Instead, other known distributed networks like the default (Buckner et al. 2008; see also Binder et al. 2009), frontoparietal control (Vincent et al. 2008), and salience networks (Seeley et al. 2007) have typically been identified within the vicinity of classical perisylvian language areas (e.g., see the 11th figure in Yeo et al. 2011). The juxtaposition of language regions near other major dominant association networks may have obscured their identification in low-dimensional, low-resolution estimates of network organization. More recently, using network parcellation approaches, Gordon and colleagues (3rd figure in Gordon et al. 2017a, 2017b; Laumann et al. 2015) and Kong et al. 2019 (see their 2nd figure) each delineated a network that matches the expected distribution of a language network that was distinct from other association networks. Nonetheless, in these schemes, the network nearest to what might be a candidate language network was given the labels “ventral attention network” and “temporal parietal” network, respectively, highlighting the uncertainty over its function.

Analyses that assume a left-lateralized language network will be present yield clear positive evidence. For example, using a clustering approach that built in priors to nudge the algorithms to identify a distinct network anchored in the left superior temporal gyrus, Lee et al. 2012 identified a candidate language network (see LAN1 in the 7th figure in Lee et al. 2012) that exhibited the hallmarks of the classic language system (i.e., containing regions in IFG, pSTC, and TP). Interestingly, this network was found to also include smaller regions distributed along the midline in dorsal posteromedial cortex (dPMC), midcingulate cortex (MCC), ventromedial prefrontal cortex, and anterior superior frontal gyrus (aSFG). This “extended” language network was recapitulated by Hacker and colleagues (2013), who used regions of activation from a meta-analysis of various cognitive domains to guide network definition (see the 5th and 7th figures in Hacker et al. 2013; see also Hampson et al. 2002). Reinforcing the role of this extended network in language, Glasser et al. (2016) (also see Ji et al. 2019) demonstrated that a similar network, defined through a multimodal approach, reveals a task-driven response during story listening. The identified regions fall at or near regions important to less domain-restricted aspects of cognitive control.

Thus, the complex literature on the network organization near to the frontal language regions likely arises, in part, because there exist multiple distinct juxtaposed networks that are simply difficult to disambiguate in group-based studies. The findings of Fedorenko et al. (2010, 2012) within individuals of spatial separation of prefrontal language regions from adjacent domain-flexible processing regions provide a compelling demonstration that a more complete description of organization may be possible when fine anatomic details are preserved.

Motivated by the opportunity to examine network organization fully within the individual, we sought to revisit and expand examination of the network organization supporting human language. Specifically, we aimed to explore the detailed anatomy of a candidate language network and contextualize it alongside other neighboring functional networks, including the default, frontoparietal control, and salience networks. What emerged is evidence that the language network is spatially distinct from other similarly organized but differentially specialized association networks. Moreover, while sharing the same organizational motif as the other higher-order association networks, regions within the language network have particularly close spatial adjacencies to a network involving motor and sensory regions hypothesized to participate in speech production and reception.

## METHODS

### Initial Experiments (Studies 1 and 2)

#### Overview.

The functional architecture of the language network of the cerebral cortex was explored using functional connectivity within individuals based on two approaches: manually selected seed-based connectivity and data-driven clustering. In all individuals tested, a clear candidate language network that occupied regions juxtaposed but distinct from other distributed association networks was observed, including the default, frontoparietal control, and salience networks. Next, data collected during a language localizer task were used to reveal language-responsive regions of the cortex. The candidate language network defined by functional connectivity overlapped in detail with regions activated by the task (Fedorenko et al. 2010). The analyses provided evidence that the extended language network, including smaller and previously underemphasized regions, responds to language task demands, supporting the idea that the distributed network is specialized for language. Data collected during a motor localizer task were used to define motor regions activating during tongue movements (*n* = 2 subjects) to explore the relationship between language network regions and sensorimotor cortices.

#### Participants.

Seven healthy adults (6 right-handed) were recruited from the greater Boston, MA, community and screened to exclude a history of neurological or psychiatric illness. Participants provided written, informed consent using procedures approved by the Institutional Review Board of Harvard University. Data were collected as part of two separate studies. In *study 1*, two subjects (ages 23 and 24; 2 females) were each scanned across 24 separate MR sessions collected over approximately 16 wk (resting-state data previously reported in Braga and Buckner 2017). Two additional potential subjects were excluded because of missing language task data and the absence of a field map. In *study 2*, five subjects (ages 20–25; 3 females) were each scanned across four MR sessions collected over 2 wk (portions of data previously reported in DiNicola et al. 2020). A sixth potential subject was excluded due to missed task trials during periods when the subject also had their eyes closed.

#### MRI data acquisition.

The detailed data acquisition protocol was previously reported in Braga and Buckner (2017) and DiNicola et al. (2020). Procedures are briefly summarized here. Data were acquired at the Harvard Center for Brain Science on a Siemens Prisma-fit 3T MRI scanner using the vendor’s 64-channel phased-array head-neck coil (Siemens, Erlangen, Germany). Subjects provided behavioral responses during the language localizer task using a custom button box. Eyes were monitored and video-recorded using an Eyelink 1000 Core Plus with Long-Range Mount (SR Research, Ottawa, ON, Canada). A four-point scale was used to record the participant’s level of alertness during each run based on the frequency and duration of eye closures. The eye video was also visually checked to flag prolonged eye closures occurring during task trials.

Blood oxygenation level-dependent (BOLD) fMRI (Kwong et al. 1992; Ogawa et al. 1992) data were acquired using a multiband gradient-echo echo-planar pulse sequence (Setsompop et al. 2012) implemented as part of the Human Connectome Project (HCP; Van Essen et al. 2013; Xu et al. 2012): TR 1,000 ms, TE 32.6 ms, flip angle 64°, 2.4-mm isotropic voxels, matrix 88 × 88 × 65, multislice 5× acceleration. Minimization of signal dropout was achieved by automatically (van der Kouwe et al. 2005) selecting a slice plane 25° from the anterior-posterior commissural plane toward the coronal plane (Mennes et al. 2014; Weiskopf et al. 2006). A rapid T1-weighted anatomic scan was acquired in each session using a multi-echo MPRAGE three-dimensional sequence (van der Kouwe et al. 2008): TR 2200 ms, TE 1.57, 3.39, 5.21, and 7.03 ms, TI 1,100 ms, flip angle 7°, 1.2-mm isotropic voxels, matrix 192 × 192 × 144 (*study 1*) or 192 × 192 × 176 (*study 2*), in-plane GRAPPA acceleration 4. A dual-gradient-echo B0 fieldmap was acquired to correct for spatial distortions: TE 4.45 and 6.91 ms with matched slice prescription/spatial resolution to the BOLD sequence.

Functional runs were flagged for exclusion if *1*) maximum absolute motion exceeded 2 mm, *2*) slice-based temporal signal-to-noise ratio was ≤135, or *3*) the value for maximum absolute motion or signal-to-noise ratio represented an outlier when values from all runs were plotted together. The raw data from flagged runs were visually checked for motion artifacts and excluded if these were deemed to be severe. All exclusions were determined prior to analysis of the task data. Following this procedure, one language localizer run was excluded for S6 due to high motion and low SNR. Furthermore, four out of 24 fixation runs and one out of eight language localizer runs were excluded for S2 after detection of signal instability (higher mean signal compared with other runs) that was later determined to arise from the gradient coil.

#### In-scanner tasks.

All seven participants provided data collected during multiple runs of a passive visual fixation task and a language localizer task (Fedorenko et al. 2010). *Study 1* also included multiple runs of a motor localizer task. Table 1 outlines the number of BOLD runs collected and included from each participant for each task. For all tasks, stimuli were projected onto a screen located behind the participant’s head and viewed through a mirror. Participants were instructed to remain still, stay alert, and stay engaged for the duration of each run. Both studies included additional tasks that were not analyzed here.

**Table 1.** No. of runs included/collected from each subject in studies 1 and 2

Subject	Study	Fixation	Language Localizer	Motor Localizer
S1	*1*	24/24	8/8	8/8
S2	*1*	20/24	7/8	8/8
S3	*2*	8/8	8/8	
S4	*2*	8/8	8/8	
S5	*2*	8/8	8/8	
S6	*2*	8/8	7/8	
S7	*2*	8/8	8/8	

*Study 1* included a fixation, a language localizer, and a motor localizer task. The numbers below each task label indicate the no. of runs included/collected. *Study 2* included a fixation and a language localizer task only. Individual runs were excluded based on criteria that included head motion and signal-to-noise ratio thresholds as well as sleepiness in the scanner. Correspondence of subject numbers shown here vs. Braga and Buckner (2017): S1 and S2 vs. S4 and S3, respectively. Correspondence of subject numbers shown here vs. DiNicola et al. (2020): S3 and S4 vs. S1 and S2; S5–S7 vs. S4–S6.

#### Fixation task.

The fixation data were used for functional connectivity definition of networks. Participants fixated a black “+” symbol presented at the center of a light gray screen. Each run lasted 7 min and 2 s. In *study 1*, data were collected over 24 MR sessions, each of which included one fixation run (total: 168 min and 48 s of fixation data per individual). In *study 2*, data were collected over four MR sessions, each of which included two fixation runs (total: 56 m and 16 s of fixation data per individual). Fixation data from *study 1* participants (S1 and S2) were previously reported in Braga and Buckner (2017) (referred to as “S4” and “S3,” respectively, in that study) but were preprocessed differently here using updated strategies to minimize spatial distortion and blurring (employed in Braga et al. 2019b).

#### Language task contrast.

Participants performed eight runs of the language localizer task developed by Fedorenko et al. (2010). This task contrasted reading meaningful sentences versus lists of pronounceable nonwords. Basic task requirements were matched between conditions (e.g., engaging with visual stimuli with the same visual features, performing button presses, and phonological processing) while preserving lexico-semantic and syntactic processing in the sentence condition (Fedorenko et al. 2010). Although the version of the language localizer task used here was not designed to capture all aspects of language (i.e., it does not include contrasts able to selectively isolate phonological and morphological components), the task contrast reveals activation in classical language regions of the IFG, pSTC, and TP as well as the cerebellum. Furthermore, the regions show spatial specificity in relation to juxtaposed regions as well as functional specificity in relation to nonlinguistic task demands (see 2nd figure in Fedorenko et al. 2011; Fedorenko et al. 2010, 2012). Both visual and auditory versions of the task identify a similar set of regions (Braze et al. 2011; Scott et al. 2017), supporting that the regions are responsive to language independent of input modality.

Participants fixated a black “+” symbol on a light gray background and read sentences (S; e.g., “TOM GOT MARRIED TO A LAWYER LAST YEAR AND SEEMED VERY HAPPY.”) and lists of pronounceable nonwords (N; e.g., “CRE ENFENTLY SILE U ALGOW OLP LENSIS ZOLLER NALD LIRM U LAS”). Sentences were presented centrally on the screen one word at a time, following and preceding presentation of the + symbol. Each sentence was composed of 12 words, with each word presented for ∼0.45 s. Each sentence was followed by a cue (an image of a finger pressing a button) lasting 0.50 s that instructed participants to press a button with their right index finger. The button response was included to keep participants engaged. Each sentence lasted ∼6.2 s.

The task began with an 18-s fixation period (+). A blocked design was used, where each condition (S or N) was presented as a block containing three consecutive trials. Four alternating blocks were presented sequentially, followed by a fixation period lasting ∼15.6 s. Twelve blocks were presented in each run. The condition order was counterbalanced across two different designs that were each performed four times by each participant, leading to eight runs collected from each participant. The designs were: 1: +, S, N, S, N, +, N, S, N, S, +, S, N, S, N, +; and 2: +, N, S, N, S, +, S, N, S, N, +, N, S, N, S, +. For the targeted contrast, the sentence conditions were contrasted with the nonwords condition (S > N; see *Task activation analyses*). The total task duration was 300 s for each run (total: 40 m of language localizer data per individual). Every sentence and nonword list presented across all sessions within an individual was unique.

#### Motor task contrasts.

To estimate regions active during tongue movements, participants performed a series of subtle controlled movements in the scanner (adapted from Buckner et al. 2011). Participants were trained before scanning on three types of movements: finger taps (sequentially touching the index and middle fingers to the thumb), foot taps (subtle dorsiflexion and plantar flexion), and tongue movements (touching the canines on the left and right side with the tip of the tongue with lips closed). Movements were made in a way that minimized muscle tension and movement of other body parts. A blocked design was employed with five active task conditions: left hand (LH), right hand (RH), left foot (LF), right foot (RF), and tongue (T) movements. Passive fixation (+) occurred between active conditions and also began and ended the run.

Each condition lasted 18 s, during which a cue stimulus (an illustration of the relevant body part) and words describing the condition (e.g., “LEFT HAND”) were shown. The cue flickered on and off on a 1-Hz cycle, and participants were cued to make the movements to the timing of the flicker. An index and a pointer finger movement were performed per cycle in the hand condition, one foot tap (dorsiflexion and plantar flexion) was performed per cycle in the foot condition, and a left and right movement were performed per cycle in the tongue condition. Participants were visually monitored while they performed these movements by the scanner operators to ensure compliance. The condition order was counterbalanced across two different designs that were each performed four times, leading to eight runs per participant. The two designs were 1: +, LH, RH, T, LF, RF, +, RF, LF, T, RH, LH, +; and 2: +, LF, RF, T, LH, RH, +, RH, LH, T, RF, LF, +. The targeted contrast was intended to isolate the orofacial motor region; hence, the tongue movement condition was contrasted with all other conditions (i.e., T > LH + RH + LF + RF; see *Task activation analyses*). The total task duration was 234 s for each run (total: 31 min and 12 s of motor localizer data per subject).

### MRI Data Processing

#### Within-subject data alignment.

Data processing procedures were previously described in detail in Braga et al. (2019b) and are summarized here. An in-house pipeline (“iProc”) optimized alignment of within-subject data collected across different scanning sessions, preserving anatomic detail as much as possible by minimizing spatial blurring and multiple interpolations (expanding on Braga and Buckner 2017; Poldrack et al. 2015; Yeo et al. 2011). Each subject’s data were processed separately. To optimize alignment, two subject-specific registration templates were created: a mean BOLD template and a T1 native-space template. BOLD data from every run contributed to the subject’s mean BOLD template, minimizing bias towards any run or session. The T1 native-space template was created by selecting a T1-weighted structural image (upsampled to 1 mm of isotropic space) that was visually deemed to have good pial and white matter boundary surface estimates (see *Projection to cortical surface*).

For each BOLD volume, three transforms were calculated to *1*) correct for head motion, *2*) correct for geometric distortions caused by susceptibility differences using a B0 fieldmap, and *3*) register the BOLD volume to the within-subject mean BOLD template. A further transform was calculated once for each subject and applied to all registered volumes that projected data from the mean BOLD template to the T1 native-space template. The four transformation matrices were composed into a single matrix that was applied to each original BOLD volume to project all data to the T1 native-space template in a single interpolation. The iProc pipeline yielded data aligned to a subject-specific template at 1-mm isotropic resolution, with minimal interpolation and signal loss.

#### Additional processing for functional connectivity.

For functional connectivity analyses, additional processing steps included regression of nuisance variables and bandpass filtering. Nuisance variables included six motion parameters plus whole-brain, ventricular, and deep white matter signal and their temporal derivatives. These signals were regressed out of native-space-projected BOLD data (using 3dTproject; AFNI version 2016.09.04.1341; Cox 1996, 2012). This was followed by bandpass filtering at 0.01–0.1 Hz (using 3dBandpass; AFNI v2016.09.04.1341; Cox 1996, 2012).

#### Projection to cortical surface.

Pial and white matter boundaries were calculated automatically using FreeSurfer’s recon-all (Fischl et al. 1999). Data were resampled from the native space to the fsaverage6 standardized cortical surface mesh (containing 40,962 vertices per hemisphere; using mri_vol2surf; Fischl et al. 1999) and then smoothed along the surface using a 2-mm FWHM kernel. Data were sampled from the gray matter at a position halfway between the white and pial surfaces using trilinear interpolation. For task-based analyses, BOLD data before nuisance regression and bandpass filtering were projected.

#### Functional connectivity analyses.

Functional connectivity analyses were performed on the surface using both seed-based and unbiased data-driven parcellation techniques. For the seed-based approach, pairwise Pearson’s product-moment correlations between the fMRI time series at each vertex were computed, yielding an 81,924 × 81,924 correlation matrix (40,962 vertices/hemisphere) for each run of BOLD data. These matrices were Fisher-transformed and averaged together, yielding a within-subject across-run mean correlation matrix with high stability for each subject. This average matrix was then inverse-Fisher-transformed back to correlation values and assigned to the vertices of a cortical template created in-house (as described in Braga and Buckner 2017). This template allowed individual vertices to be selected for real-time visualization of the resulting correlation maps using the Connectome Workbench’s wb_view software (Marcus et al. 2011). For final visualization of seed-based connectivity maps, correlation values were converted back to *z*(*r*) using the Fisher transform.

#### Initial observation and hypothesis.

The observation that a candidate language network may be detectable within individuals was made during a previous exploration of the functional anatomy of the default network in S1 (referred to as “S4” in Braga and Buckner 2017). While manually selecting seeds, a distinct network was observed that followed the distributed motif of other association networks (i.e., it resembled the canonical default and frontoparietal control networks in that it contained regions distributed in frontal, parietal, and temporal association cortices) but occupied spatially distinct regions of the cortex. Notably, the network contained large regions in the left lateral temporal and left inferior frontal cortices near classical language areas. The hypotheses were formed that *1*) the anatomic details of this candidate language network could be reproducibly defined in additional subjects and *2*) the network would show increased activity during a task broadly targeting linguistic processes. We targeted these hypotheses using network mapping techniques and by comparing the network maps with regions activated during a language localizer task (Fedorenko et al. 2010). Critically, whereas the initial discovery of the network was made using manual procedures, the observations were converged upon by automated classification.

#### Manual targeting of candidate language network.

**Fig. 1.**
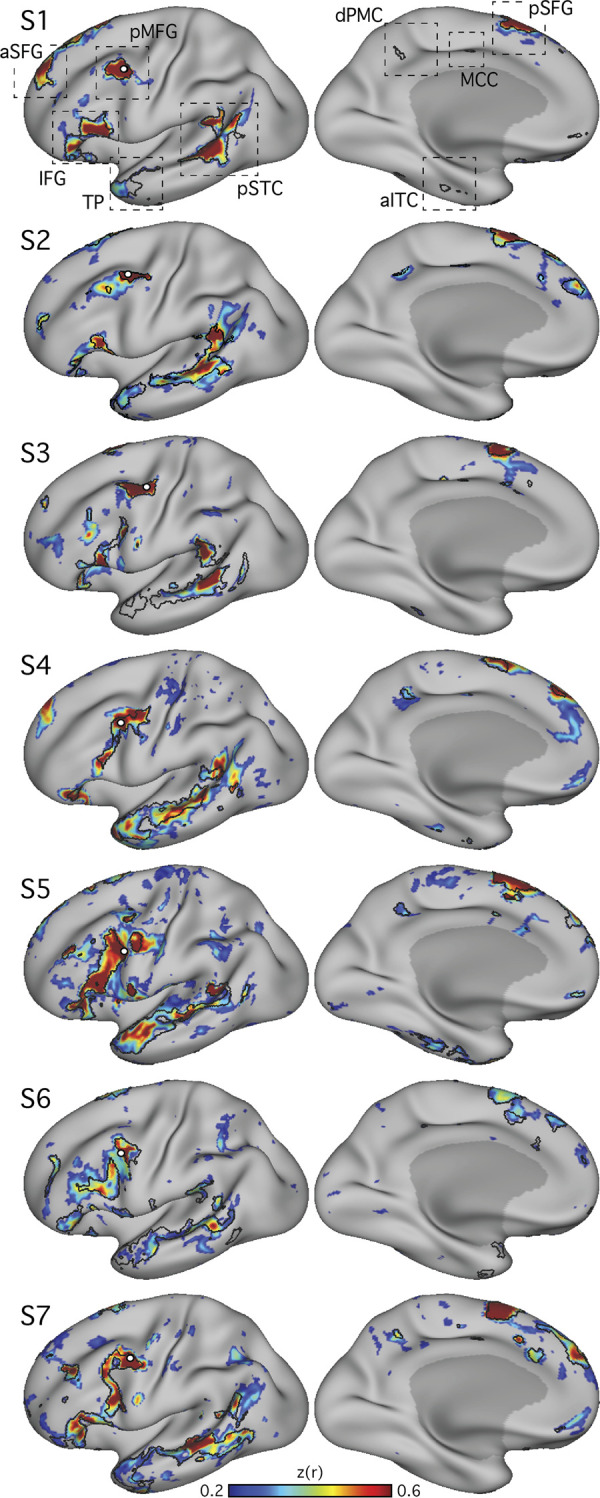
Within-individual intrinsic functional connectivity identifies a candidate-distributed language network. Seven subjects (S1–S7) each reveal a candidate language network. Seed regions (○) are displayed at or near the posterior middle frontal gyrus (pMFG). Correlation patterns are shown on an inflated cortical surface representation of the left hemisphere. In each panel, the candidate language network defined by data-driven parcellation (see Fig. 4) is shown in black outline. In each subject, the correlation patterns (color bar) show a network that included regions located near classical language regions of the inferior frontal gyrus (IFG; Broca’s area) and posterior superior temporal cortex (pSTC; Wernicke’s area). The network also revealed regions distributed across multiple cortical zones (see dashed boxes at *top*), including the posterior superior frontal gyrus (pSFG), the anterior superior frontal gyrus (aSFG; appearing in medial and/or lateral portions in different subjects), and the temporal pole (TP). Smaller regions observed consistently in 5 or more subjects included the dorsal posterior medial cortex (dPMC), the middle cingulate cortex (MCC), and the anterior inferior temporal cortex (aITC). Lateral (*left*) and medial (*right*) views are shown. *z*(*r*), Fisher’s *r*-to-*z* transformed Pearson’s product-moment correlations.

To identify the candidate language network in additional subjects, seed regions were manually selected from the left prefrontal cortex at or near to where the precentral sulcus meets the posterior middle frontal gyrus (i.e., pMFG; Fig. 1). This brain region was targeted because it contained a prominent region of the candidate language network in the initial exploration of S1 (see also Fedorenko et al. 2010; Glasser et al. 2016; and Lee et al. 2012). An iterative process similar to that described in Braga and Buckner (2017) and Braga et al. (2019b) was used for seed selection. A seed vertex was identified in each individual that revealed a robust network with a spatial distribution that resembled the candidate language network, as initially observed in S1. Correlation maps were thresolded at *z*(*r*) > 0.2 for visualization and displayed with the Jet look-up table (colorbar) set to a range between 0.2–0.6, ignoring negative values. A network was deemed robust if it generally revealed high correlation values [*z*(*r*) ≈ 0.6], but also if the network regions displayed sharp boundaries surrounded by areas of low correlations [*z*(*r*) ≈ 0.2]. Specifically, to assure that the candidate language network was being detected selectively, the observer’s knowledge of spatial features from other networks was also used in seed selection. For example, candidate seed vertices were not selected if they revealed prominent connectivity to the posterior midline at or near the cingulate and retrosplenial cortices, which are hallmark features of the default network. Similarly, candidate seed vertices revealing patterns resembling the frontoparietal control network were not selected. In other words, the seed-selection process targeted specific features of the initially observed candidate language network and excluded features of other known networks.

#### Confirmation of the network from distributed cortical zones.

**Fig. 2.**
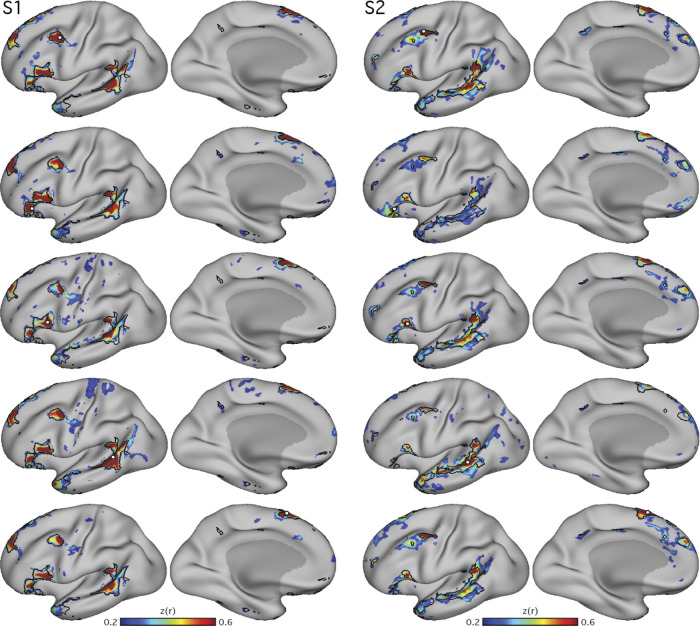
Distributed organization of the candidate language network is confirmed using seed regions in multiple cortical locations. In 2 subjects (S1 and S2), seed regions (○) were selected from different portions of the network identified in Fig. 1. In each panel, the candidate language network defined by data-driven parcellation (see Fig. 4) is shown in black outline to provide landmarks for comparing across panels. In each subject, seed regions were placed in the inferior frontal gyrus at an anterior (2nd row from *top*) and posterior site (3rd row from *top*) as well as in the posterior superior temporal sulcus (4th row from *top*) and posterior superior frontal gyrus (*bottom* row). Although the maps differ in their details, the large-scale distribution and location of the network regions are appreciably similar across seed regions, with regions of high correlation falling generally within the parcellation-defined boundaries. *z*(*r*), Fisher’s *r*-to-*z* transformed Pearson’s product-moment correlations.

To determine whether the network was spatially selective and similar if defined outside of prefrontal cortex, additional seed regions were examined in two subjects (S1 and S2). The approximate locations of regions revealed by the original pMFG seeds were targeted in the anterior and posterior IFG, the pSTC, and the posterior superior frontal gyrus (pSFG; Fig. 2). In each zone, for each subject, the iterative seed selection process was again followed, resulting in a single seed that targeted the candidate language network in each cortical zone and subject. A similar network was detectable from seeds in all zones.

#### Generalization of the candidate language network across acquisition task states.

**Fig. 3.**
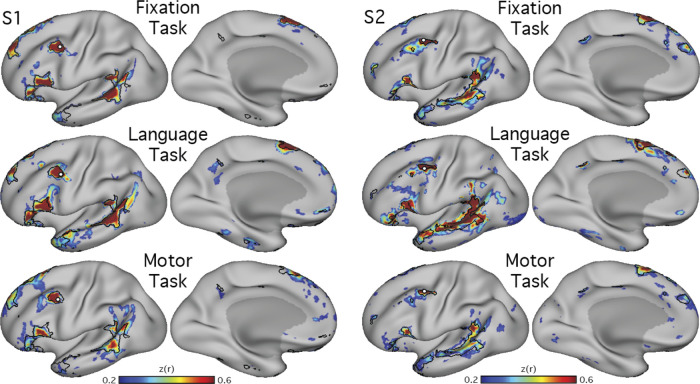
The connectivity-defined candidate language network generalizes across data acquired in different task states. Functional connectivity reliably defined the candidate language network across 3 distinct tasks, showing that the presence of the network is not dependent on a specific cognitive context (see text for task descriptions). Note that the location of the seed region (○) was optimized for each data set to show that the topography of the network is stable despite minor differences in functional shifts that might occur due to task context. Note also that the optimal seed location can vary across data sets even when collected during the same task context (see examples of within-state variance in 3rd supplemental figure in Braga and Buckner 2017 and 3rd figure in Braga et al. 2019b). *z*(*r*), Fisher’s *r*-to-*z* transformed Pearson’s product-moment correlations.

To explore whether the detection of the candidate language network was dependent on the behavioral state of participants during data acquisition, functional connectivity was performed using data acquired during three different tasks. Data from the fixation, language localizer, and motor localizer tasks were analyzed separately for two subjects (S1 and S2). For each task, in each subject, initially the same seed vertex as previously selected from the fixation data (see *Manual targeting of candidate language network*) was used. If this seed failed to produce a robust map in the other two task datasets, another seed was selected at or near the pMFG following the iterative process described above. A similar network was detectable using data from all three tasks (Fig. 3). We have previously found that the “ideal” vertex for targeting a given network can change across different groupings of runs, even when all data are acquired during the same task. This could be due to small differences in alignment of data from different runs.

#### Data-driven parcellation.

**Fig. 4.**
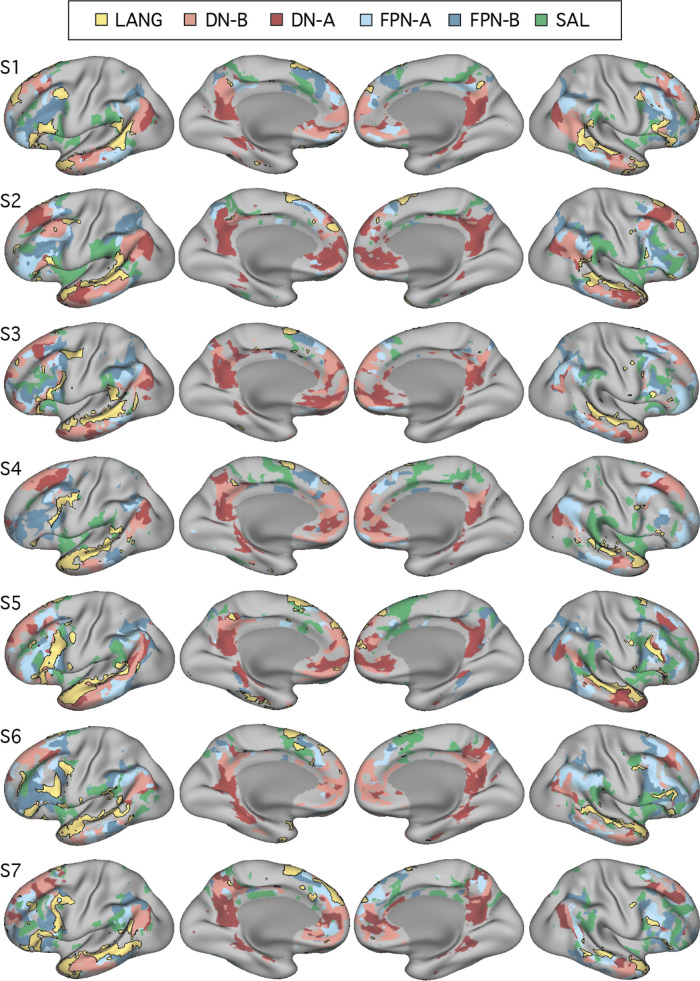
Close juxtaposition of the candidate language network with neighboring distributed networks revealed by data-driven parcellation. *K*-means clustering was used to parcellate the cortex into 17 discrete networks. The candidate language network (LANG; yellow and black outline) was observed in all participants (S1–S7). Network regions were recapitulated in all of the nine zones highlighted in Fig. 1, including a region in the temporal pole that extended rostrally. Further regions can also be observed in the right hemisphere. From the parcellation solutions, 5 additional networks were selected for further analysis due to their spatial proximity to the language network and their identification within classic language regions in prior data-driven network analyses (e.g., see Yeo et al. 2011). These networks were the salience network (SAL; green), frontoparietal control network-A (FPN-A) and -B (FPN-B) (blue), and default network-A (DN-A) and -B (DN-B) (red). The LANG network had a complex spatial relationship with these neighboring networks, showing regions closely packed with default, frontoparietal control, and salience network regions in the temporal cortex and inferior and dorsal frontal cortices. The *left* 2 columns show lateral and medial views of the inflated left hemisphere, whereas the *right* 2 columns show the right hemisphere.

Although seed-based correlation is able to reveal the topography of the intrinsic networks, it relies heavily on observer input, which could result in bias. To confirm that the definition of the candidate language network was not a consequence of observer bias, a data-driven parcellation analysis was performed for each subject using *k*-means clustering. Preprocessed BOLD data from the fixation task were concatenated in time, and MATLAB’s *kmeans* function (version 2015b; MathWorks, Natick, MA) was used to cluster the time series into networks. Default settings were used (1 random initialization, 100 iterations, squared Euclidean distance metric). As the results will reveal (Fig. 4), similar network estimates were found for both the seed-based and parcellation approaches, suggesting that the identification of the candidate language network is robust to different network discovery methods. *K*-means clustering was performed in each individual at *k* = 17 (as in Yeo et al. 2011) for the initial experiments (*studies 1* and *2*). It is important to note that the clustering and seed-based approaches yield similar but not identical network estimates and that the network topography is influenced by the number of clusters defined. In *studies 1* and *2*, *k* = 17 was used because it produced a network that was found to correspond to the candidate language network as defined by seed-based connectivity, and it recapitulated other previously observed distinctions between distributed networks (Braga and Buckner 2017) in all individuals. In a post hoc analysis conducted after all experiments had been run, we tested the effect of changing *k* in two individuals. The LANG network was found to be stable over a range of dimensionalities; however, it was lumped with other networks around or below *k* = 7 and was split into separate networks around and above *k* = 26.

#### A priori selection of networks.

In order to explore language-driven task responses in relation to the spatial distributions of multiple closely juxtaposed association networks, five networks in addition to the candidate language (LANG) network were selected for further analysis from the 17-network parcellation. The selected networks included the two networks previously identified within the canonical default network (DN-A and DN-B), two networks that are positioned near to the canonical frontoparietal control network (FPN-A and FPN-B; see Braga and Buckner 2017), and the salience network (SAL; Dosenbach et al. 2007; Seeley et al. 2007). The networks were identified and labeled according to previously described anatomic features (Braga and Buckner 2017; Dosenbach et al. 2007). Anatomic details of FPN-A and FPN-B were previously reported for two subjects (including S1, labeled “S4” in Braga and Buckner 2017).

As can be seen in Fig. 4, the networks differed in their detailed anatomy across subjects. Specific spatial relationships served as useful anchoring points, but given the complex relationships of the networks, any assignment must be considered a hypothesis awaiting independent functional confirmation to build confidence (such as provided for DN-A and DN-B in DiNicola et al. 2020 and sought here for the LANG network). That said, certain features and patterns are largely consistent across subjects. Both FPN-A and FPN-B occupy regions of the lateral inferior frontal cortex and parietal regions at or near the intraparietal sulcus. Within the inferior parietal lobule, FPN-A typically occupies a region more ventral to FPN-B and more anterior to DN-B. Even so, these regions are heterogeneous and difficult to match across subjects. Perhaps the most reliable identifying feature is that the DN-A, DN-B, FPN-A, and FPN-B networks follow a stereotyped anterior-posterior sequence along the inferior lateral temporal cortex. The relative position in this portion of the brain of FPN-A (anterior) and FPN-B (posterior) served as a useful guide for labeling those networks. In all subjects, one of the 17 networks defined by clustering was deemed to correspond with each of the DN-A, DN-B, FPN-A, and FPN-B networks based on these previously reported anatomic features.

The SAL network was identified by the presence of regions in the anterior inferior parietal lobule or supramarginal gyrus, in the inferior frontal cortex and insula, and a region or set of regions along the dorsal midline, sometimes circling the medial somatomotor cortex in a “U” shape (Fig. 4). The similar large-scale distribution of the SAL network regions across subjects offers some confidence that the same broad network was being targeted. For example, note that the parietal region of the SAL network was located in the supramarginal gyrus, anterior to FPN-A, in all subjects. However, the correspondence was not perfect, with gaps evident between the SAL network parietal region and the other network regions in some subjects. In each subject, the network that most closely followed the anatomy of the canonical SAL network was chosen.

In this way, five additional distributed association networks that were all near the LANG network regions were identified a priori. These networks were each tested for task-driven response during the language localizer task contrasts.

#### Task activation analyses.

Data were analyzed for task-driven response using the general linear model as implemented by FSL’s FEAT (Woolrich et al. 2001). Preprocessed and smoothed data from each BOLD run were entered into a first-level analysis. Surface-projected data from the left and right hemispheres were analyzed separately, and the results were combined after for visualization and a priori-defined network activation analysis. The data and model were high-pass filtered using a cutoff of 100 s to reduce the influence of low-frequency noise. A linear term was included in the model to account for linear drifts in the data. Each task condition was modeled as a separate explanatory variable using a block design (see *In-scanner tasks*). The explanatory variables were convolved with a double-γ-hemodynamic response function. Temporal derivatives were included in the model to account for variations in the hemodynamic response. In the language localizer task, for the targeted contrast of sentences > nonwords conditions, at each vertex the β-value for the nonword condition was subtracted from the β-value for the sentences condition. In the motor localizer task, for the contrast of tongue movements > other movements, at each vertex the β-value for the tongue condition was multiplied by 4, and the sum of the β-values for the right hand, left hand, right foot, and left foot conditions were subtracted. For both contrasts, the resulting values at each vertex were converted to *t*-statistics by dividing by their standard error and then converted to a *z*-statistic. Within each subject and task, the *z-*statistic maps from all runs were averaged together using *fslmaths* (Smith et al. 2004).

**Fig. 5.**
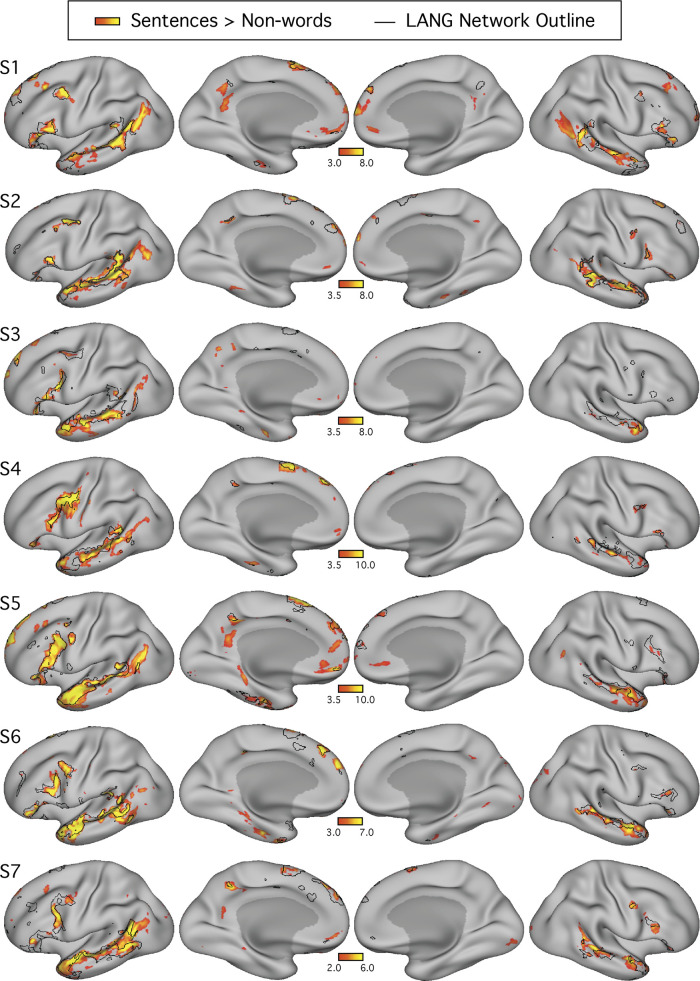
The candidate language network shows close spatial correspondence with regions activated during a language task contrast. The language network (LANG) is shown in black outline and was defined using *k*-means clustering. Independently acquired data collected during a language localizer task contrast (Fedorenko et al. 2010) reveals cortical response to linguistic demands. Red-yellow color bars show within-individual *z*-normalized β-values (i.e., “increased activation”) for the contrast of reading sentences vs. reading lists of nonwords. In all subjects (S1–S7), the language task activations fell largely within the boundaries of the intrinsically defined candidate language network. The overlap was not perfect, and in some cases hints of other networks can be seen (e.g., see S1 and S5), although these exceptions were not consistent across subjects. The upper and lower thresholds were selected by eye for each subject to show the distribution of language-responsive regions, while removing regions showing low responses. The detailed anatomy of the distributed intrinsic network corresponds closely with regions showing task-driven activation, including in smaller areas extending beyond the classical language zones (e.g., see S2 and S4), suggesting that the entire intrinsically organized network is functionally specialized.

For visualization, *z*-thresholds were selected to best demonstrate the task activation patterns for each subject (see colorbar limits in Fig. 5). A lower threshold was picked just above that needed to remove low-confidence activations (i.e., small, randomly dispersed spots or speckles showing low *z* values), and an upper threshold that allowed vertices of low and high activation within the contiguous regions to be discerned was picked. Similar maps were obtained when the same lower threshold (>3.0) was used for all subjects (data not shown).

**Fig. 6.**
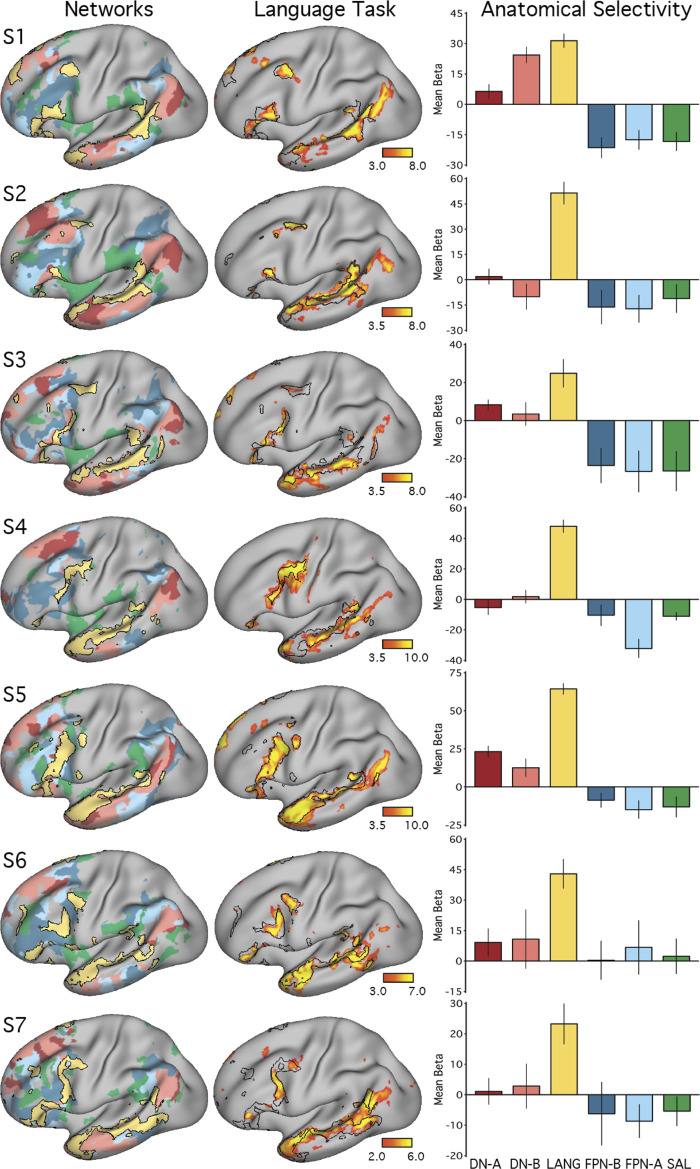
The candidate language network is selectively activated during a language task contrast. *Left*: the networks defined by intrinsic functional connectivity from Fig. 4 are replotted. The candidate language network (LANG) is shown in yellow, with the salience network (SAL) in green, the frontoparietal control networks (FPN-A and FPN-B) in blue, and the default networks (DN-A and DN-B) in red. *Middle*: task activation for the contrast of reading sentences vs. reading lists of nonwords (sentences > non-words) is shown, with the intrinsic LANG network outline in black (see Fig. 5 for other views). *Right*: bar graphs show the mean β-values for the sentences > nonwords contrast, averaged within each within-individual a priori-defined network, along with the standard error of the mean. Despite differences across individuals, LANG was the only network showing consistently higher activation for sentences > non-words, showed the highest activation of all networks in all participants, and in some cases (S2, S4, and S7) was the only network that showed clear increased activity in the task contrast.

A key question was whether the topography of the task contrast map for the language localizer task corresponded to the topography of the intrinsic connectivity LANG network. To address this question, two approaches were used. First, the maps were visually compared; the borders of the spatial map from the parcellation analysis were overlaid onto the cross-run average task activation map (Fig. 5). Second, a network-of-interest approach was used using the six a priori selected networks defined in each subject (see *A priori selection of networks*). The average β-value for the contrast of sentences > nonwords was calculated for all vertices falling within each network. Values from both the left and right hemispheres were included. Average β-values were calculated for each run of the language localizer task, leading to eight estimates of the network’s recruitment during the task for each network and subject (except for S2 and S6, who each provided 7 runs; Table 1). The cross-run average β-value for each network was then plotted in a bar graph, along with the standard error of the mean (Fig. 6). This latter analysis has the benefit that there are no thresholds or subjective steps; the magnitude and variance of the response in each data-driven, a priori-defined network are obtained and quantified in each individual.

### Prospective Replication (Study 3)

#### Overview.

*Study 3* was conducted as a prospective replication after the results of *studies 1* and *2* were known and reported in a preprint (Braga et al. 2019a).

#### Participants.

Six healthy adults (ages 19–29, 5 right-handed, 4 females) were recruited to complete four scanning sessions each. All were screened to exclude neurological or psychiatric illness, and all provided written, informed consent through a protocol approved by the Institutional Review Board of Harvard University. For all *study 3* participants, fixation data and results from additional tasks were previously reported (2nd experiment in DiNicola et al. 2020). Most *study 3* procedures were the same as for *studies 1* and *2*, with differences detailed below.

#### MRI data acquisition, in-scanner tasks, and data processing.

MRI data acquisition methods matched those described for *study 2*. Exclusion criteria were also carried forward, but with a slightly stricter maximum motion cutoff of 1.8 mm (as per the 2nd experiment in DiNicola et al. 2020). As in *studies 1* and *2*, exclusions were determined prior to analysis. Three fixation runs were excluded for S11 and one for S12 due to high motion.

In-scanner tasks included the visual fixation task (11 total runs, 77 min and 22 s of fixation data per individual) and the language localizer task (6 total runs; 30 min of language data per individual), with the same parameters as described for *studies 1* and *2*. Data were collected across four MR sessions. Three fixation runs and two language localizer runs were collected during each of the first three sessions, and two additional fixation runs were collected during the fourth session. No runs of the motor localizer task were acquired. One participant (S10) discontinued participation after two sessions, resulting in fewer runs of the fixation (42 min and 12 s) and language (20 min) tasks for this individual. Table 2 shows the number of BOLD runs collected and included for each participant in *study 3*.

**Table 2.** No. of runs included/collected from each subject in studies 3 and 4

Subject	Study	Fixation	Language Localizer	Motor Localizer
S8	*3*	11/11	6/6	
S9	*3*	11/11	6/6	
S10	*3*	6/6	4/4	
S11	*3*	8/11	6/6	
S12	*3*	10/11	6/6	
S13	*3*	11/11	6/6	
S14	*4*	6/6	4/4	
S15	*4*	5/11	6/6	
S16	*4*	11/11	6/6	
S17	*4*	11/11	6/6	
S18	*4*	11/11	6/6	

*Studies 3* and *4* included the fixation and language localizer tasks. The numbers below each task label indicate the no. of runs included/collected. Two subjects (S10 and S14) completed only 2 sessions, resulting in fewer collected runs of each task. Individual runs were excluded based on criteria that included head motion and signal-to-noise ratio thresholds as well as sleepiness in the scanner. For S15, 1 fixation run was included, in error, that surpassed the motion threshold (>1.8 mm). Data from additional tasks were previously reported for these subjects. Correspondence of subject numbers shown here vs. DiNicola et al. (2020): S8–S16 vs. S7–S15; S17 and S18 vs. S17 and S18.

MRI data processing procedures, including within-subject alignment through iProc, functional connectivity processing, and cortical surface projection, were preserved from *studies 1* and *2*. Of note is that data from two participants (S8, S9) were processed following Freesurfer and system software upgrades (i.e., from Freesurfer version 4.5.0 to version 6.0.0 and from centOS6 to centOS7). Extensive testing revealed minimal differences between data preprocessed on the original versus upgraded systems, and since all analyses were conducted within subjects, minor differences would not influence results.

#### Prospective replication of functional connectivity analyses.

Functional connectivity procedures were carried forward from *studies 1* and *2*. In *study 3*, for the data-driven parcellation, *k*-means estimates were calculated for solutions between *k* = 10 and *k =* 20, and the solution featuring the fewest clusters that differentiated the six distributed networks (Fig. 11) was chosen for each individual (similar to the 3rd experiment in DiNicola et al. 2020). For three individuals, the chosen *k* matched that from a prior experiment, for which differentiation of only two networks (DN-A and DN-B) was prioritized (2nd experiment in DiNicola et al. 2020). For the other three individuals (S8, S11, and S13), increasing *k* to differentiate all six networks resulted in only minimal differences to DN-A and DN-B. For S11, referential features of the FPN-B network, which could be clearly observed using the seed-based approach, were observed in two different clusters, one predominantly in the left hemisphere and the other in the right. Both clusters were included in the FPN-B estimate. Critically, networks were defined prior to task activation analyses.

#### Prospective replication of task activation analyses.

All procedures used to obtain the task activation results for *studies 1* and *2* were applied to the data from *study 3*. Briefly, general linear model parameters were preserved, and for each individual a mean *z-*statistic contrast map for the sentences > nonwords contrast was thresholded for visualization and comparison to the LANG network estimate (Fig. 12). The network-of-interest approach was then used to quantify the recruitment of six selected networks by the language localizer task contrast (Fig. 13).

### Prospective Triplication (Study 4)

#### Overview.

*Study 4* was conducted as a prospective triplication. The minor procedural differences introduced in *study 3* were maintained in *study 4*.

#### Participants.

Five additional healthy adults (ages 19–22, all right-handed, all female) were recruited to complete four scanning sessions each. As in *studies 1–3*, all participants were screened to exclude neurological or psychiatric illness and provided written, informed consent through a protocol approved by Harvard University’s Institutional Review Board. Fixation data and results from additional tasks were also previously reported for participants in *study 4* (3rd experiment in DiNicola et al. 2020). Data from a sixth potential participant were excluded before analysis due to high motion and a presentation error, which affected multiple runs of the language localizer task.

#### MRI data acquisition, in-scanner tasks, and data processing.

MRI data acquisition and processing methods, as well as exclusion criteria, were identical to those described for *study 3*. Exclusions were determined before task analysis. Six fixation runs were excluded for S15 due to motion, and one run with a maximum motion above the 1.8-mm threshold was included for this individual, in error. Because network estimation had already been performed for this individual when this error was noticed, we retained this run to avoid potential bias.

For each participant, 11 runs of the visual fixation task (77 min and 22 s of total data) and six runs of the language localizer task (30 min of total data) were collected. No runs of the motor localizer task were acquired. One participant (S14) discontinued participation after two sessions, resulting in fewer runs of the fixation task (42 min and 12 s) and language localizer (20 min; see Table 2).

iProc was used for data processing and template alignment, and additional processing procedures for functional connectivity and surface projection matched those described for *studies 1–3*. Data from all individuals in *study 4* were processed following the software and system upgrades described in *study 3*.

#### Prospective triplication of functional connectivity and task activation analyses.

Functional connectivity procedures were the same as those detailed for *study 3*. For all participants in *study 4*, the chosen *k* matched that from a prior publication (3rd experiment in DiNicola et al. 2020). To identify the full set of networks, one individual (S17) required higher *k* than was used in *studies 1* and *2*, but only minor differences in features of the LANG network were observed at higher versus lower *k*. For S18, referential features of the FPN-A network were observed in two clusters, one per hemisphere; both clusters were included in the FPN-A estimate. As in *studies 1–3*, the networks were defined before any task analyses.

All task activation analysis procedures for quantifying network recruitment by the language localizer contrast, described in detail for *studies 1* and *2*, were again applied, without any iterative adjustments, to the data from *study 4*.

#### Composite analyses.

After the replication and triplication analyses, data from all individuals were combined to further explore network properties.

#### Network lateralization analyses.

Left lateralization is a commonly noted feature of the language system. As will be described, across *studies 1–4*, in most subjects the LANG network showed greater representation in the left hemisphere. To quantify this potential lateralization, in comparison with that of five other networks, we used two metrics. For each participant, using the network estimates derived from the *k*-means clustering, we first calculated the percentage of vertices in each hemisphere (excluding medial wall vertices shown in dark gray) that a network occupied:

$$\text{No}\text{. of vertices}\;\left(\%\right)=\frac{\text{no}\text{. of network vertices in hemisphere}}{\text{total no}\text{. of vertices in hemisphere}}\times 100$$

Next, within each network, we calculated the relative number of vertices per hemisphere:

$$\text{Lateralization}=\frac{\text{left hemisphere vertices}-\text{right hemisphere vertices}}{\text{left hemisphere vertices}+\text{right hemisphere vertices}}$$

For this metric, similar to in prior work (Binder et al. 1995; Mahowald and Fedorenko 2016), positive values corresponded to left lateralization. For both metrics, group means were plotted and compared (Figs. 21 and 22).

In addition, although greater LANG surface area was expected in the left hemisphere than in the right, we predicted that task activation in each hemisphere would be similar. More specifically, even with relatively fewer vertices in the right hemisphere, LANG network regions in both hemispheres were predicted to exhibit similar levels of recruitment for the language localizer task. To test this, β-values were extracted from within the bounds of each network using the within-subject average sentences > nonwords contrast for each hemisphere. Group mean β-values were then calculated and plotted (Fig. 21, *bottom*).

Note that for two subjects, S11 and S18, the data-driven clustering produced estimates of FPN-B and FPN-A, respectively, that split these networks into two clusters, one in each hemisphere. These two clusters were then grouped together. In a confirmatory analysis, the group means of the lateralization indices and the task activation values (Fig. 21) were also computed with exclusion of these two subjects, which showed very similar results (not shown).

Finally, individual-specific lateralization data were plotted to better characterize networks that showed three different surface area patterns: left-lateralized (LANG), right-lateralized (FPN-A), and bilateral (DN-A). These plots allowed for assessment of pattern consistency across individuals as well as identification of rarer contralateralization in specific participants (e.g., S15 showed a right lateralized LANG pattern; Fig. 22, *top*).

### Experimental Design and Statistical Analysis

This series of studies includes *n* = 18 participants, two of which were scanned over 24 MRI sessions and the remainder of which were scanned over four sessions. In all analyses, data were averaged over all usable runs that were collected from each individual (see Tables 1 and 2). An initial cohort of *n* = 7 participants was collected, analyzed, and reported in a preprint publication (Braga et al. 2019a). Prospective replication (*n* = 6) and triplication (*n* = 5) cohorts were then collected and analyzed. Most analyses focused on within-individual quantities, but following all individual-focused analyses a set of composite analyses were performed on pooled data, shown in Figs. 21 and 22. Functional connectivity between brain regions was calculated in MATLAB (version 2015b; http://www.mathworks.com; MathWorks, Natick, MA) using Pearson’s product moment correlations and Fisher’s *r*-to-*z* transformation before averaging across runs. Network parcellation was performed using MATLAB’s *kmeans* function (version R2015b). Task data were analyzed using the general linear model as implemented using FSL’s first-level FEAT (Woolrich et al. 2001). The cross-run average task activation map was created by taking the *z*-normalized β-maps from each run and then averaging together using *fslmaths* (Smith et al. 2004).

## RESULTS

### Studies 1 and 2

#### A candidate language network is identified by functional connectivity within the individual.

The language network (LANG) was defined in all seven individuals tested from *studies 1* and *2* (Fig. 1), using seeds manually placed in the pMFG. In all cases, a distributed network that contained regions within the IFG, the pSTC, the TP, and the pSFG was observed. The pSTC region sometimes extended into the inferior parietal lobule near to the supramarginal gyrus, but a clear and robust region in angular gyrus was not observed (see Figs. 1, 2, and 4). The LANG network contained further regions, extending to upwards of nine cortical zones in the left hemisphere (highlighted in Fig. 1) replicating the extended language network defined by Lee et al. (2012) and Hacker et al. (2013) (see also Glasser et al. 2016). A distinct region in the left anterior superior frontal gyrus (aSFG; appearing in medial and/or lateral portions in different subjects) was observed in all subjects. Regions in the dorsal posteromedial cortex (dPMC; at or near the posterior cingulate and precuneus), the middle cingulate cortex (MCC), and the anterior inferior temporal cortex (aITC) were observed in five subjects. In four subjects (S1, S4, S5, and S7), suggestion of a further region was observed at or near the ventromedial prefrontal cortex despite this region suffering from signal dropout. The presence of a network region in each of the nine highlighted zones in Fig. 1, replicated across a majority of individuals, suggests that the candidate language network is widely distributed and extends beyond regions that define the classical language system.

#### The candidate language network generalizes across data sets and analysis methods.

To support that the identified regions formed a distributed interconnected network, seeds were placed in four of the other large regions of the LANG network. In each case, the seeds produced correlation maps that were similar to that defined by the original pMFG seed (Fig. 2), suggesting that definition of the LANG network was not dependent on a single seed location or vertex.

A further analysis tested whether the definition of the LANG network was dependent on the specific task that was performed during data acquisition. To address this question, data were analyzed from the same individuals during the performance of two additional tasks: the language and motor localizer tasks. In both cases, intrinsic connectivity from a seed in the pMFG revealed a similar distribution of regions as that identified using the visual fixation task data (Fig. 3). Subtle differences were observed. For instance, the correlations were generally higher, and the defined regions slightly larger, during the language task in *subject S2*. Similarly, in *subject S1*, the LANG region in the TP was emphasized in the language task data compared with the other tasks, and the pSTC region extended further into the angular gyrus. One possible explanation is that these differences could be a consequence of larger signal fluctuations driven by the demands of the language task. Despite these differences, the same general distribution of regions was revealed across the three task contexts, including the active motor tasks. In most cases there was clear correspondence in the location and shape of network regions across the different tasks (Fig. 3).

The final analysis ensured that the definition of the LANG network was not a result of observer bias in the selection of seed regions. A data-driven parcellation approach to defining the networks (*k*-means clustering) was performed. In all participants, parcellation revealed a candidate language network (Fig. 4) with near-complete overlap with the network as defined by seed-based connectivity (see black border outlines in Fig. 1), including smaller distributed regions (Figs. 1–3; see especially S1, S3, and S7 in Fig. 1). An interesting difference was that in the temporal pole the clustering approach revealed a large region that was diminished or absent in the thresholded seed-based maps. The temporal pole suffers from signal dropout in MRI due to magnetic susceptibility differences with the nearby sinuses. It is possible that the parcellation approach is able to better define networks in regions of low signal because it clusters all vertices based on their relative pattern of correlations, rather than using an absolute correlation threshold.

#### The candidate language network is bilateral but left-lateralized.

In addition to the left-hemisphere regions detailed above, the LANG network also displayed multiple distinct regions in the right hemisphere (Fig. 4). The locations of these regions were roughly homologous to the zones observed in the left hemisphere, with a similar distributed organization, including the right pMFG, IFG, pSTC, pSFG, and TP in all subjects. Both hemispheres contained a large region spanning almost the length of the superior temporal sulcus. However, for other regions the right-hemisphere homologs were visibly smaller in surface area (Fig. 4). In zones where evidence was found for small regions in the left hemisphere (pPMC, MCC, aITC), the homologous right-hemisphere regions were sometimes not observed.

It is important to note that the parcellation approach simultaneously clusters all surface vertices across both hemispheres. Hence, the apparent left-right asymmetry in size observed in the clustering solution likely reflects actual differences in the network topology, as opposed to a spatial bias that can occur in seed-based approaches by selecting seeds from the left hemisphere. As a confirmation that the observed asymmetry was not a result of such bias, when seed regions were placed in the right pSTC region (biasing the correlations toward the right hemisphere) in some subjects, the functional connectivity patterns revealed a similar distribution of regions that were also larger on the left than on the right (data not shown).

#### The candidate language network is similarly organized and closely juxtaposed with other association networks.

In all subjects from *studies 1* and *2*, the LANG network contained regions distributed in multiple zones of association cortex with a broad organizational pattern that paralleled other distributed association networks like the default and frontoparietal control networks (Fig. 4). In other words, if two networks had regions positioned side by side in one association zone, they likely also contained side-by-side regions in other association zones. Moreover, the spatial sequence of networks, from LANG to DN-B to DN-A (yellow-pink-red networks in Fig. 4), can be observed in multiple distributed zones in each individual. Clear examples can be seen in temporal and parietal cortices but also along posteromedial cortices, where the LANG network contains a small region in the dPMC neighboring the large regions characteristic of the default network (see S1, S2, S4, S5, and S7 in Fig. 4). Within the IFG, regions of DN-B and LANG networks were closely interdigitated, occupying alternating regions curving along the inferior edge of the left IFG in a caudal to rostral axis (see S1, S2, S3, and S5 in Fig. 4 for clear examples). Along the pSTC, DN-B and LANG regions were also closely situated with complex demarcations, in some cases along the length of the superior temporal sulcus (S1, S2, S5, and S7 in Fig. 4). In some cases, DN-A regions also bordered LANG regions, for instance, near the left IFG (see S2 and S5 in Fig. 4), the left TP (S2, S3, and S5), the left pSTC (S5 and S6), and left dPMC (S5 and S7).

The LANG network also bordered the frontoparietal control networks in multiple (but not all) locations. In the IFG, several subjects displayed close-knit LANG and frontoparietal control network regions, particularly FPN-B (see S1, S5, S6, S7 in Fig. 4). LANG and FPN regions were closely positioned along the midline near the pSFG, which also contains a characteristic frontoparietal control network region (e.g., see Fig. 2 in Vincent et al. 2008). The LANG network also bordered the salience (SAL) network near the anterior inferior parietal lobe close to the sylvian fissure and supramarginal gyrus, as well as in posterior regions of the IFG near or in BA6. However, the parietal FPN-A and FPN-B regions did not consistently border the LANG network at or near the pSTC region.

The overall picture was that language regions were distinct but positioned near to separable association networks, with consistent neighboring relationships across individuals that were evident in multiple cortical locations.

#### The candidate language network responds to language task demands.

Figure 5 shows the boundaries of the LANG network in each individual, defined by the unbiased data-driven parcellations, overlaid onto regions showing task activation during a language task contrast collected from the same individuals. In each subject, the spatial similarity can be clearly observed between the two maps, one defined by functional connectivity and one by relative increases in activity during reading sentences compared with lists of nonwords. For each individual, a threshold was selected by eye to allow the topography of regions showing strong and weak task effects to be observed, respecting that data quality is not equivalent in all subjects. No masking of the task activation maps that might accentuate their similarity with the intrinsic connectivity maps was applied.

The resulting maps revealed three key findings (Fig. 5). First, in all subjects the regions showing strong task effects were largely confined to the boundaries of the intrinsically defined candidate language network (but see descriptions of exceptions below). Second, in many places the regions showing task activation had boundaries that occurred at the boundary of the intrinsic LANG network regions. As a particularly striking example, note that in S5 the task-activated regions, particularly in the IFG and lateral temporal cortex, almost entirely fill in the spaces between the boundaries of the LANG network. Other clear examples include the left pSTC regions in S1 and left lateral frontal and temporal regions in S6. Third, the association between task activation and intrinsic connectivity was typically not restricted to one part of the brain. Instead, evidence of task activation was found in intrinsic network regions distributed across all nine zones highlighted in Fig. 1, particularly when all subjects are considered together. For example, note the small dPMC region of the LANG network in S2, S4, and S7 or the multiple regions on the right-hemisphere lateral surface in S2, S4, S6, and S7. The importance of this is that it suggests that the whole distributed network is recruited during the language task contrast, even smaller regions predicted by functional connectivity, rather than just the classical perisylvian language regions.

The overlap was not perfect. Regions of clear task activation that did not overlap with the intrinsic network could be observed in some subjects. For example, in addition to the LANG network regions, the task activation map for S5 (Fig. 5) revealed midline regions along the retrosplenial and posterior cingulate cortices, the anterior medial prefrontal cortex, and a circumscribed region of the medial temporal lobe in an organization reminiscent of DN-A (Braga and Buckner 2017; Braga et al. 2019b). Importantly, the evidence for the recruitment of this other system was restricted to few subjects (S5 and possibly also S1 and S7), whereas the evidence for a close association between language task activation and the intrinsic candidate language network was evident in all subjects. One possible exception was the left angular gyrus, which showed strong task-driven activation in multiple subjects (e.g., S1, S2, and S5; Fig. 5) but did not seem to contain a region of the LANG network defined by intrinsic connectivity in any subject.

To quantitatively test for the selectivity of task-driven responses, the average language task activation effect (mean β-value) was calculated within each of the six networks (LANG, DN-A, DN-B, FPN-A, FPN-B, and SAL) defined a priori using functional connectivity (Fig. 6). In all subjects, the intrinsic LANG network showed the highest level of activation during the language task. In most subjects, the LANG network showed selectivity, being activated considerably more than all other networks. In some subjects (e.g., S2, S4 and S7 in Fig. 6), the LANG network was the only network showing activity clearly above baseline. These observations suggest that the LANG network is selectively recruited during the present task contrast targeting semantic and syntactic processing (although note the regional exceptions outlined in the previous paragraph). In contrast, neighboring networks showed limited if any evidence of activation despite their close spatial proximity in multiple cortical zones. One exception was DN-B, which showed a strong task activation effect in *subject S1*; however, this observation did not generalize to other subjects. In S5, DN-A also showed evidence of a response that was not found across subjects.

#### The language network abuts an intermediate network that is adjacent to tongue motor and auditory regions.

The proximity of Broca’s area to motor representations of the tongue, lips, and other oral structures in the inferior portion of the motor strip has been noted previously (Geschwind 1970; Krubitzer 2007). Given the possibility of delineating neighboring functional regions with precision in individuals, we explored the relationship between the language network and sensory and motor regions important for hearing and vocalization. The language network defined in the present set of individuals contained two frontal regions, one in the IFG and one in the pMFG, that were close to the motor strip along the central sulcus (Figs. 1, 4, and 5; see also Fedorenko et al. 2010 and Glasser et al. 2016). In addition, a large extended region belonging to the LANG network was located in the temporal cortex near the auditory cortex along the supratemporal plane. We initially explored the relationship between these LANG regions and the nearby anatomy in the two subjects that provided a motor localizer task (S1 and S2).

**Fig. 7.**
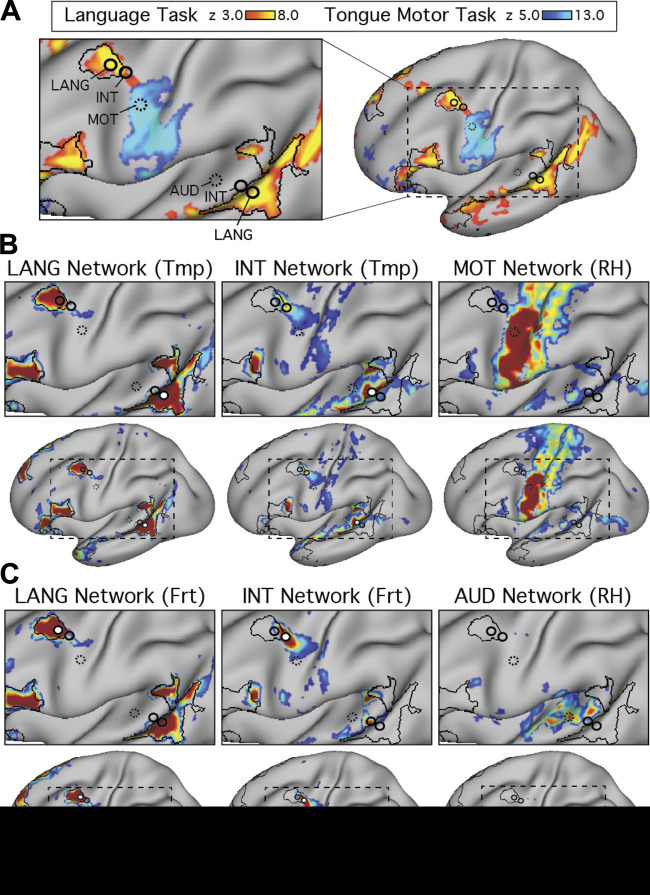
Distributed networks link language regions with tongue motor and auditory regions in S1. An intermediate network (INT) was observed, which sits in between the language network (LANG) and both the temporal auditory (AUD) and frontal orofacial motor (MOT) regions. *A*: yellow regions show activations during the language localizer task (as in Fig. 5; sentences > nonwords), whereas blue regions show regions displaying increased response during a separate tongue movement task contrast (tongue movements > hand and foot movements) provided by the same subject. The black outline displays the parcellation-defined intrinsic language network (LANG; Fig. 4). Solid open circles are centered on seed vertices that were used to define intrinsic connectivity networks in the remaining panels. *B* and *C*: seed-based intrinsic connectivity patterns from seeds selected from the temporal (Tmp; *B*) and frontal lobes (Frt; *C*). Auditory and motor regions were recapitulated using functional connectivity using seed regions placed in the contralateral [right (RH)] hemisphere, as correlation patterns close to the seed are difficult to interpret. Dashed open circles refer to the reflected location of the contralateral seeds. White-filled circles denote the location of the seed used to define correlation patterns in that panel. The INT network displays an organization that parallels the LANG network, containing neighboring regions in both inferior frontal and temporal cortices as well as along the posterior superior frontal midline (not shown). The function of the INT network is unclear; however, its distributed organization and juxtaposition with LANG, MOT, and AUD regions in multiple locations suggests that it may form part of a hierarchy linking language and sensorimotor functions. Task activations are shown as mean *z*-normalized β-values and intrinsic correlations as Fisher’s *r*-to-*z* normalized Pearson’s product-moment correlations, ranging from 0.2 to 0.6, as in Fig. 1.

**Fig. 8.**
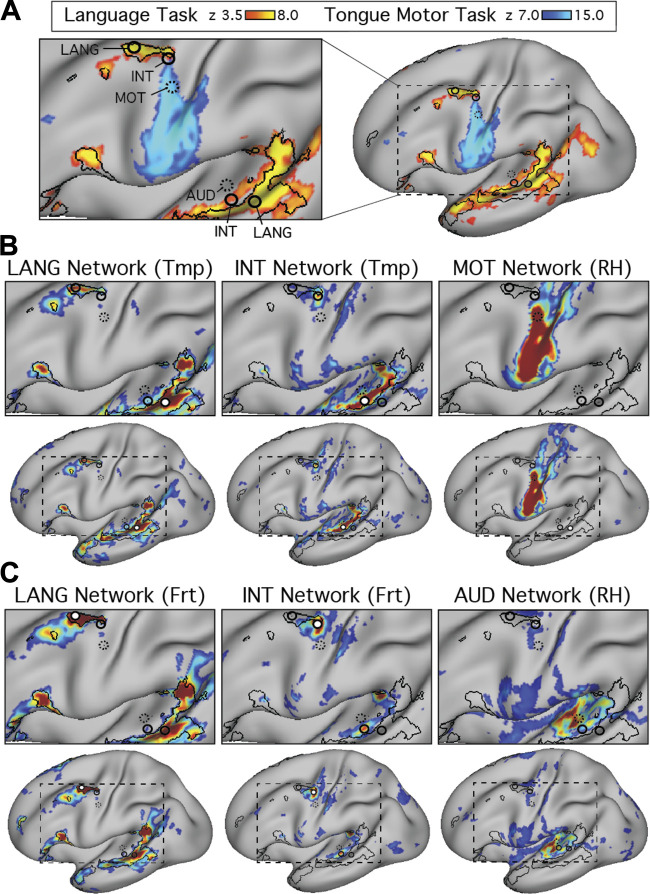
Distributed networks link language regions with tongue motor and auditory regions in S2. Generalizing the findings from S1 (Fig. 7), intrinsic connectivity in S2 also revealed an intermediate (INT) distributed system that bridged the spaces between the language network (LANG) and sensorimotor regions for hearing (AUD) and tongue movements (MOT). *A*: task-activated regions are shown for the language (yellow) and tongue motor localizer (blue) task contrasts. *B* and *C*: seed-based intrinsic connectivity patterns from seeds selected in the temporal lobe (Tmp; *B*) and the frontal lobe (Frt; *C*) as well as in homologous regions of the right hemisphere (RH). Task activations are shown as mean *z*-normalized β-values and intrinsic correlations as Fisher’s *r*-to-*z* normalized Pearson’s product-moment correlations, ranging from 0.2 to 0.6, as in Fig. 1.

We began by mapping orofacial motor and separately auditory regions. Tongue motor regions, defined using the motor localizer task, occupied inferior portions of the central sulcus and nearby gyri (blue regions in Figs. 7*A* and 8*A*; see also Brown et al. 2008; Carey et al. 2017; Hesselmann et al. 2004). Functional connectivity from a seed placed in the central sulcus on the contralateral hemisphere revealed a bilateral motor network (MOT; Figs. 7*B* and 8*B*). A close correspondence was observed in both subjects between the intrinsic connectivity MOT network and task-driven activations (also see the 6th figure in Gordon et al. 2017b). To define auditory sensory regions, a seed was placed in the contralateral hemisphere on the supratemporal plane at or near Heschl’s gyrus. This defined an auditory network (AUD; Figs. 7*C* and 8*C*) based on intrinsic connectivity that comprised a bilateral set of circumscribed regions at the approximate anatomic location of Heschl’s gyrus, and no other clear regions of high correlation anywhere on the cortical surface, in both subjects. No auditory localizer was available for these subjects, so the function of the AUD network was presumed based on the bilateral supratemporal distribution of the regions.

We next mapped the immediately adjacent zones of the cortex. We hypothesized that the language network regions in the lateral frontal cortex would be juxtaposed with the tongue motor region (Krubitzer 2007) and that the temporal regions would be juxtaposed with the auditory regions. Instead, we unexpectedly observed a small gap between sensorimotor (MOT and AUD) networks and the LANG network near the pSTC and pMFG and a larger gap in the IFG (Figs. 7 and 8). When seed regions were placed in the spaces between these networks, we identified a smaller, intermediate network (INT). The INT network occupied regions in between the LANG network and the MOT and AUD networks in both frontal and temporal lobes and also contained a small region neighboring the LANG region in the pSFG (observable in medial view; not shown). Both subjects displayed a similar distribution of the INT network. Notably, in the frontal lobe the INT network bridged the space between tongue regions and the pMFG LANG region, forming a LANG-INT-MOT sequence of regions. The IFG region did contain a neighboring INT network region (clear in S1 in Fig. 7, less clear in S2 in Fig. 8); however, this was separated from the tongue region by the salience network in these subjects (see SAL network in IFG in Fig. 4). Along the midline, a LANG-INT-MOT sequence could also be seen extending from rostral to caudal regions near the pSFG in both subjects. In the superior temporal cortex, the sequence of LANG-INT-AUD networks occurred in two separate places, one more caudally near or at the planum temporale and one more rostrally nearer Heschl’s gyrus (Figs. 7*C* and 8*C*).

**Fig. 9.**
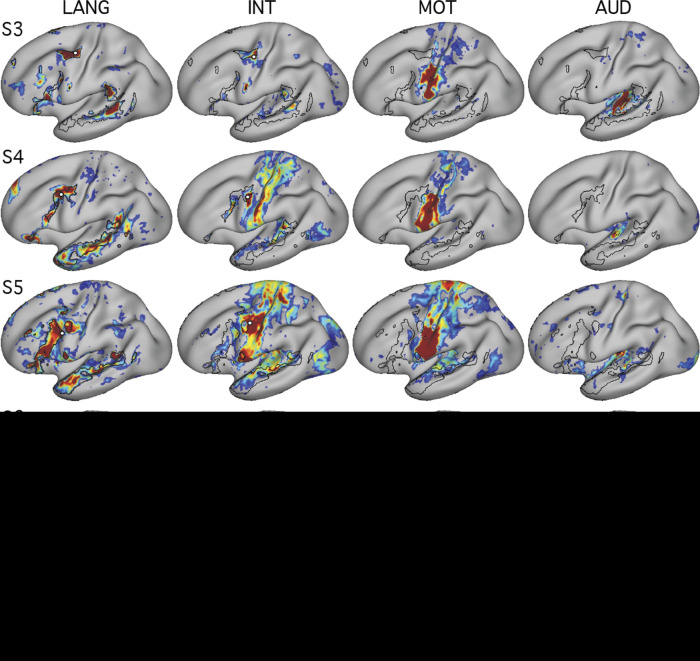
Distributed networks link language regions with tongue motor and auditory regions in S3–S7. Generalizing the findings from S1 (Fig. 7) and S2 (Fig. 8), intrinsic seed-based connectivity was used to confirm the presence of language (LANG), intermediate (INT), motor (MOT), and auditory (AUD) networks for the remaining subjects (S3–S7) from the original cohort (*studies 1* and *2*). Black outlines display the parcellation-defined intrinsic language network (Fig. 4). White-filled circles denote the location of the seed used to define correlation patterns in that panel. Dashed circles refer to the reflected location of contralateral seed locations. A network that included key features of regions following the expected distribution of the INT network as identified in *subjects S1* (Fig. 7) and *S2* (Fig. 8) could be defined in all subjects, but with varying degrees of separation from nearby networks.

**Fig. 10.**
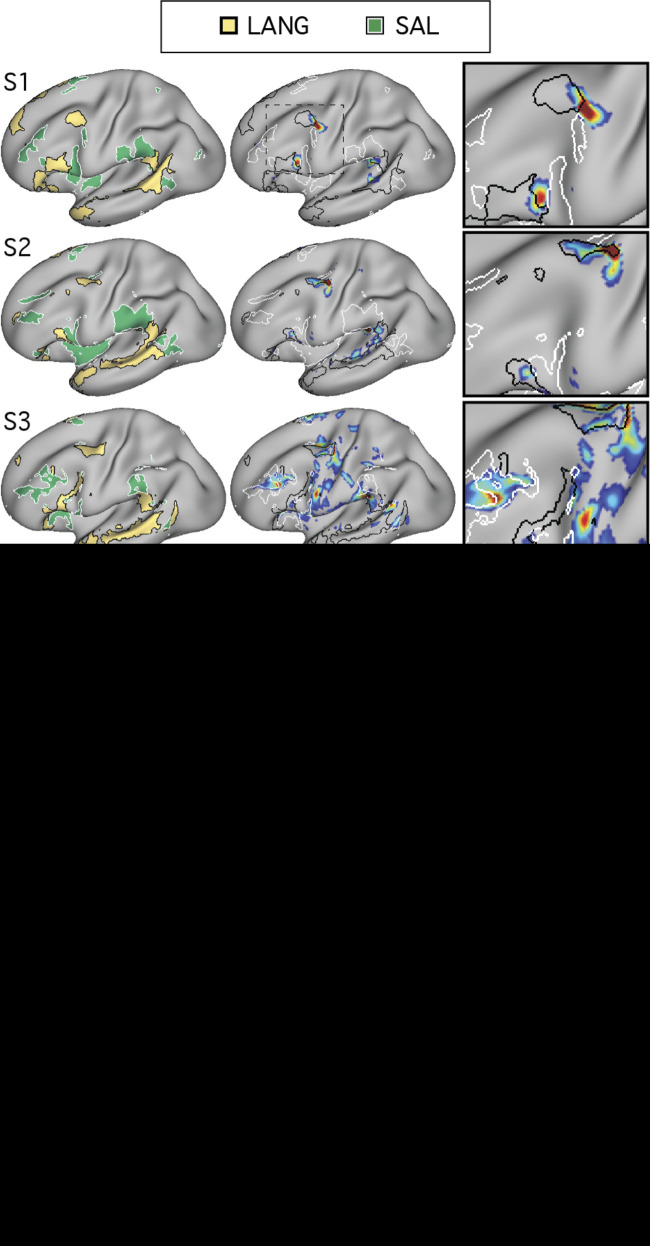
Details of spatial relationships between the intermediate (INT), language (LANG), and salience (SAL) networks. In lateral frontal cortex, SAL was closely positioned next to regions of the LANG and INT networks. Closer analysis of these 3 networks showed that the INT network was more often positioned next to the LANG regions than SAL regions. INT regions typically extensively bordered the LANG network, were located next to LANG regions in all subjects, and were not consistently juxtaposed with SAL regions to the same extent (e.g., see S2 and S7).

The initial observation of the INT network and description of its relationships to nearby regions was based on analyses of the two subjects that provided the most data (S1 and S2) and provided data collected during a motor localizer task. After publication of initial findings from these two subjects (Braga et al. 2019a), we defined the INT, MOT, and AUD networks in the remaining subjects. Replicating procedures used in *subjects S1* and *S2*, a manually selected seed-based approach was used to define the INT network based on seeds placed in the pMFG (Fig. 9), pSFG (Fig. 10), and lateral temporal cortex (not shown). Key referential features of the INT network were observed in all individuals: *1*) the presence of regions in lateral temporal cortex, situated in between LANG and AUD networks; *2*) the presence of regions in inferior frontal cortex, closely positioned next to LANG regions in pMFG; and *3*) the presence of a region in pSFG, just posterior to the LANG network region there. The sequence of LANG-INT-AUD regions in temporal cortex could be observed in each subject using seeds placed in the frontal cortex (Fig. 9). However, a clear separation of INT regions from nearby networks was not uniformly achieved. The clearest separation was achieved in S6, where the INT network occupied distinct regions from LANG, MOT, and AUD networks. In S3 and S7, the INT network overlapped with restricted portions of the LANG network. In S4 and S5, the INT network was distinct from LANG regions; however, it also showed correlation with other sensorimotor regions. Better separation of INT and motor regions was achieved using a pSFG seed in S4 (Fig. 10; see also replication and triplication analyses in Figs. 14 and 19).

The small IFG region of the INT network was not juxtaposed with MOT network regions but rather appeared to be positioned next to regions of the SAL network, leading to the appearance of a LANG-INT-SAL sequence. To further explore the relationship between the INT and SAL networks, we visualized the INT network, defined using seed regions in pSFG, along with the borders of the SAL and LANG networks (Fig. 10). The distal pSFG seed allowed the topography of the INT network to be appreciated within the inferior frontal cortex. In some subjects, the INT network did not border the SAL network near the pMFG region (see S2, S6, and S7 in Fig. 10). In many cases, the INT region in pMFG extensively bordered the LANG network and only partly bordered SAL (see *subjects S3* and *S4* in Fig. 10). We also did not observe a single case where the INT network bordered the SAL network in pMFG but not the LANG network. A similar observation was made for the IFG region, with the possible exception of S3 (Fig. 10).

### Study 3

#### Definition of the language network replicates across a second set of individuals.

**Fig. 11.**
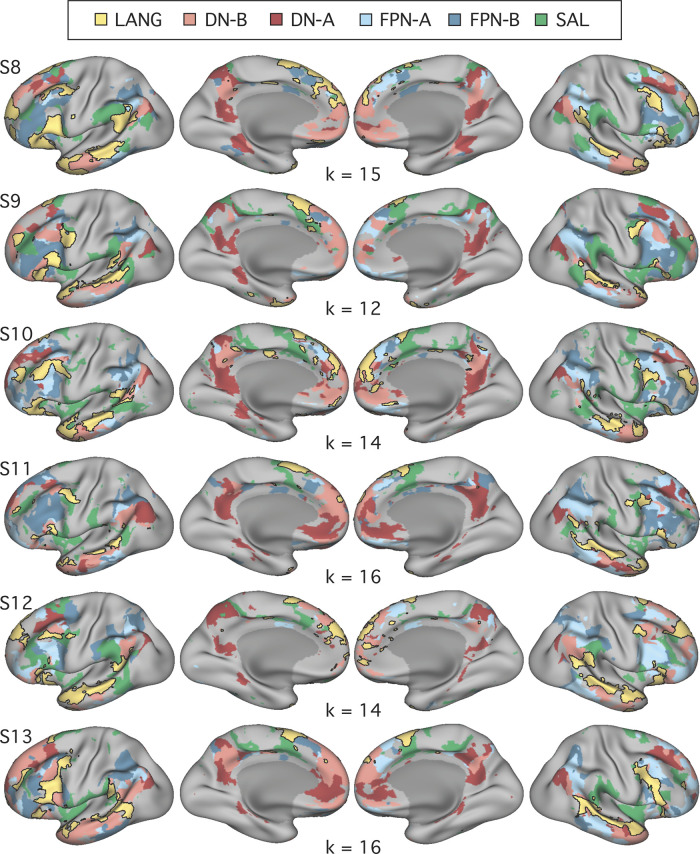
Replication of close juxtaposition of the language network with neighboring distributed networks revealed by data-driven parcellation. *K*-means clustering was used to parcellate the cortex into *k* networks in each individual from the replication cohort (*study 3*). Confirming the results from the original cohort (*studies 1* and *2*; Fig. 4), the language network (LANG; yellow and black outline) was observed in all participants (S8–S13). When all subjects were considered, network regions were recapitulated in all of the 9 zones highlighted in Fig. 1. From the parcellation solutions, 5 additional networks were selected for further analysis: the salience network (SAL; green), frontoparietal control network-A (FPN-A) and -B (FPN-B) (blue), and default network-A (DN-A) and -B (DN-B) (red).

Six subjects (S8–S13) were analyzed as part of a prospective replication of the findings from *studies 1* and *2*. Network definition procedures were repeated from *studies 1* and *2*, with minor differences (see *Prospective replication of functional connectivity analyses*). A candidate language network could be defined in all six subjects using both seed-based (Fig. 14) and data-driven clustering approaches (Fig. 11). In all subjects, the LANG network contained prominent regions in the left pMFG, IFG, pSTC, and pSFG. Regions could also be seen in all subjects in the TP, aSFG, and MCC. Of the remaining nine zones described in the initial experiments (Fig. 1), three of the six subjects showed a region in dPMC (S8, S10, and S12), and only one subject showed the region in aITC (S9). Three subjects showed a region in the putative ventromedial prefrontal cortex zone (S8, S10, and S12). In the seed-based connectivity maps (lateral view shown on Fig. 14; medial view not shown), hints of smaller regions were observed in additional subjects in the aITC (S10, S12, and S13), dPMC (S13), and ventromedial prefrontal cortex (S13). All subjects also showed right-hemisphere homologs of the more prominent left-hemisphere regions, including smaller regions in the dPMC and MCC in five subjects (S8, S10, S11, S12, and S13).

The juxtaposition of the DN-B and LANG networks was also replicated in *study 3*, with their interdigitation along IFG present in four subjects (S9, S11, S12, and S13; Fig. 11) and complex patterns evident in the lateral temporal cortex. The sequence of LANG-DN-B-DN-A was evident in the parietal lobe in four individuals (S8, S10, S11, and S13; Fig. 11). Note that in S9 and S12, the selected data-driven clustering solution contained a third cluster of regions within the default network along the midline, which left a gap in the midline and lateral parietal lobe between the networks deemed to be most representative of DN-A and DN-B. It is possible that the observation of this third cluster, which has been made in a minority of subjects, is a consequence of blurring between two networks within the idiosyncratic anatomy of a minority of subjects.

DN-A regions did not seem to border LANG regions in multiple locations in this cohort (*study 3*), as observed in the original cohort (*studies 1* and *2*). Juxtaposition of LANG and FPN-B was again observed in the IFG and pSFG. In the anterior inferior parietal lobe and temporoparietal junction, LANG and SAL regions were juxtaposed in all six subjects.

#### Replication of language network response to language task demands.

**Fig. 12.**
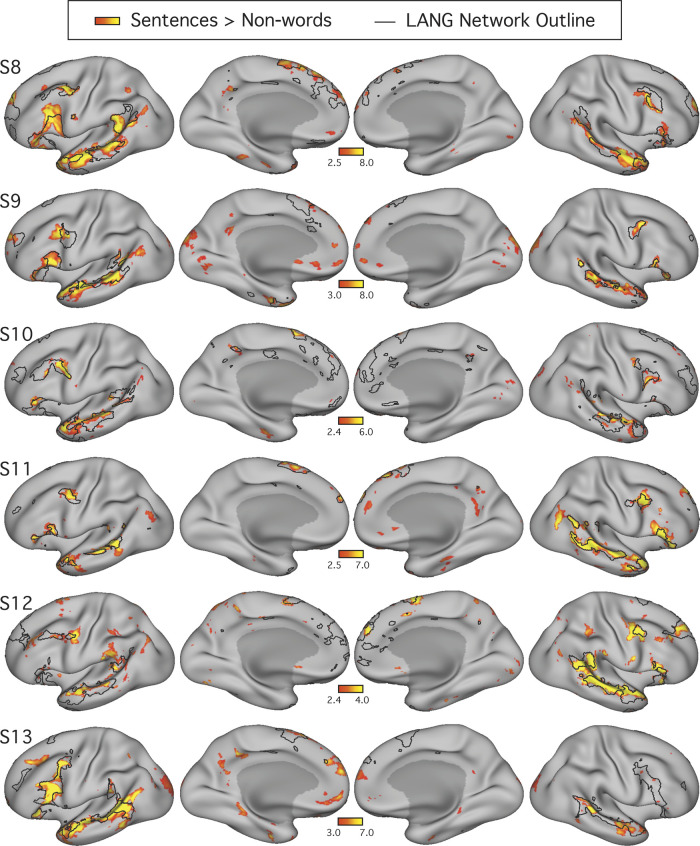
Replication of close spatial correspondence between the language network and regions activated during a language task contrast. Analysis of the replication cohort (*study 3*) recapitulated the findings from the original cohort (*studies 1* and *2*; see Fig. 5). The language network (LANG) is shown in black outline and was defined using *k*-means clustering (Fig. 11). In all subjects (S8–S13), the language task activations fell largely within the boundaries of the intrinsically defined candidate language network. The detailed anatomy of the distributed intrinsic network corresponds closely with regions showing task-driven activation, including in smaller areas extending beyond the classical language zones (e.g., see S8 and S11), suggesting that the entire intrinsically organized network is functionally specialized. The overlap was not perfect, and in some cases hints of other networks can be seen (e.g., see S13), although these exceptions were not consistent across subjects. Unusually, in 2 subjects, S11 and S12, the task activation maps revealed larger regions in the right than in the left hemispheres. These subjects were also found to have unusually bilateral or slightly right-lateralized LANG networks when the relative size of regions in each hemisphere was later compared (Fig. 22).

Figure 12 shows the boundaries of the LANG network defined by clustering, overlaid onto the activation map for the task contrast of reading sentences > nonword lists. The main findings from *studies 1* and *2* were broadly replicated. First, regions showing task activation were largely confined to the boundaries of the intrinsic connectivity network (see S11 and S13 for good examples and S10 for a bad example). *Subject S12* had poor correspondence on the left hemisphere but good correspondence and more widespread activation on the right. Second, in many instances the border of the LANG network again coincided with the boundaries of the task-activated regions (see S9, S10, S11, and S13 in Fig. 12). Third, evidence for activation within the LANG network was found throughout the distributed network, including smaller regions in dPMC (S8 and S10), aITC (S8, S9, S10, S11, and S13), and ventromedial prefrontal cortex (S8, S9, and S13), as well as right hemisphere regions. The MCC region was a notable exception, with no evidence of activation at the threshold selected.

Some of the exceptions noted in the original cohort were also replicated here. A prominent region of activation was observed in the angular gyrus in S9, and perhaps S13, with hints also detected in S8, S11, and S12. Hints of the DN-A network were also observed along the midline in some individuals (S9 and S13).

**Fig. 13.**
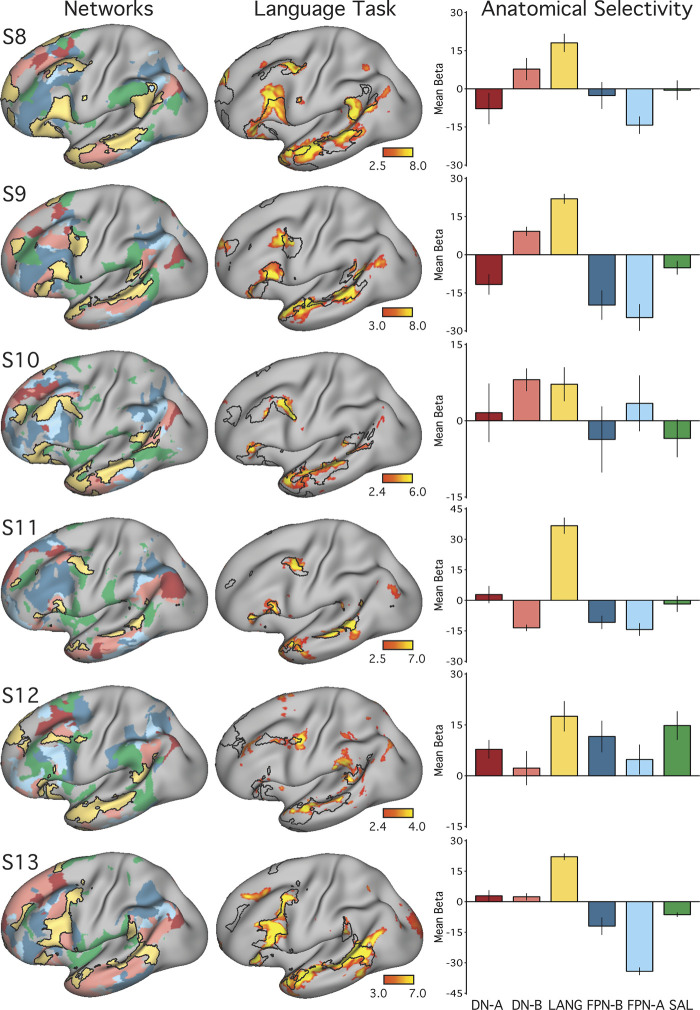
Replication of selective activation of the language network during a language task contrast. Analysis of the replication cohort (*study 3*) recapitulated the findings from the original cohort (*studies 1* and *2*; see Fig. 6). *Left*: the networks defined by intrinsic functional connectivity from Fig. 11 are replotted. The candidate language network (LANG) is shown in yellow, with the salience network (SAL) in green, the frontoparietal control networks (FPN-A and FPN-B) in blue, and the default networks (DN-A and DN-B) in red. *Middle*: task activation for the contrast of reading sentences vs. reading lists of nonwords is replotted from Fig. 12. *Right*: bar graphs show the mean β-values for the sentences > nonwords contrast, averaged within each within-individual a priori-defined network, along with the standard error of the mean. Despite differences across individuals, LANG was the only network showing consistently higher activation for sentences > nonwords and in some cases (S11 and S13) was the only network that showed clear increased activity in the task contrast.

The average language task activation effect (mean β-value) was calculated within the a priori defined networks for the *study 3* cohort (Fig. 13). The finding of selectivity for language task responses in the LANG network was replicated. In all subjects except S10, the LANG network showed the highest average task response. In a majority of subjects, the LANG network showed considerably higher activation than the other networks. In two subjects (S11 and S13), LANG was the only network showing activation above baseline. In three subjects (S8, S9, and S10), DN-B also showed evidence of task engagement. In one subject (S12), three other networks showed evidence of recruitment. With these exceptions, the other networks showed limited evidence of task recruitment, with FPN-A and FPN-B showing evidence in a majority of subjects for reduced activation for the task contrast employed.

#### Replication of observation of intermediate network across a second set of individuals.

**Fig. 14.**
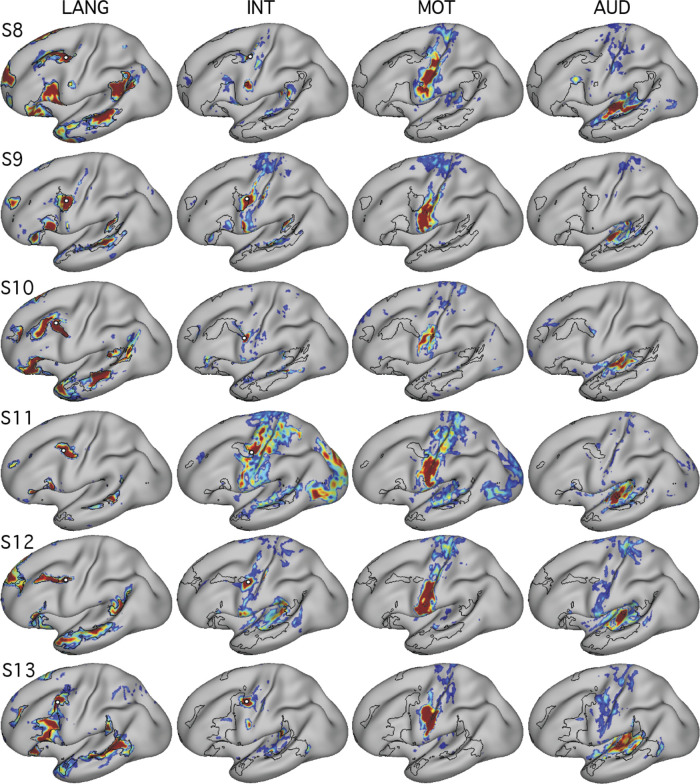
Replication of distributed networks linking language regions with tongue motor and auditory regions. Generalizing the findings from *studies 1* and *2* (see Figs. 7, 8, and 9), intrinsic seed-based connectivity was used to confirm the presence of language (LANG), intermediate (INT), motor (MOT), and auditory (AUD) networks for the replication cohort (S8–S13; *study 3*). Black outlines display the parcellation-defined intrinsic language network (see Fig. 11). White-filled circles denote the location of the seed used to define correlation patterns in that panel. Dashed circles refer to the reflected location of contralateral seed locations. A network that included regions following the expected distribution of the INT network as identified in subjects S1 (Fig. 7) and S2 (Fig. 8) could be defined in all subjects, but with varying degrees of separation from nearby networks (e.g., see S13 vs. S11).

**Fig. 15.**
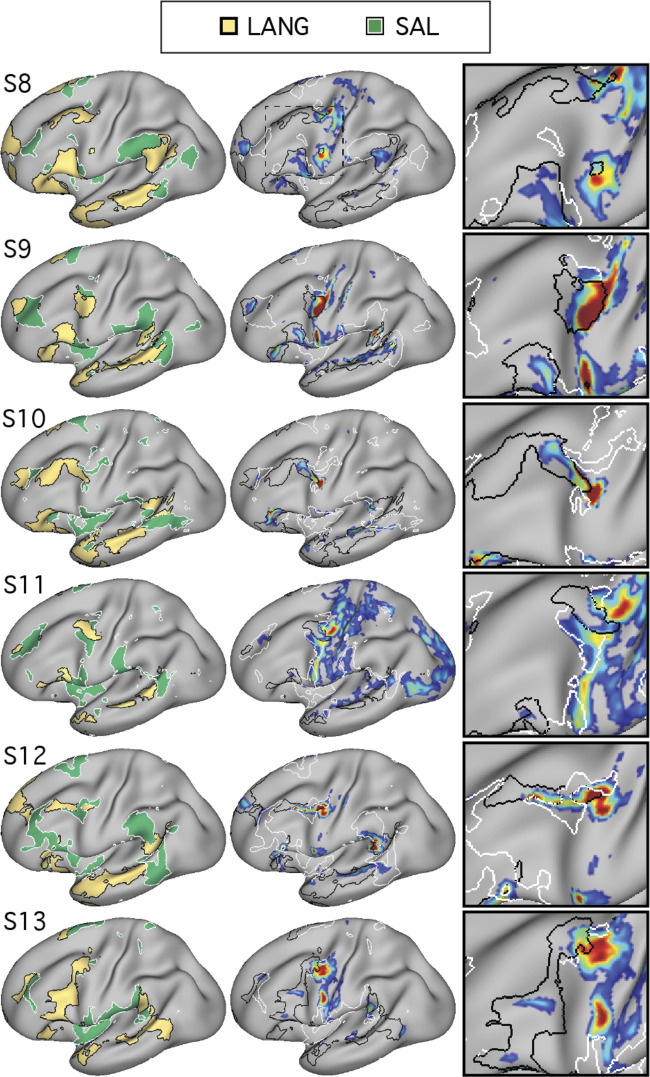
Replication of spatial relationships between the intermediate (INT), language (LANG), and salience (SAL) networks. Replicating the results from the original cohort (*studies 1* and *2*; see Fig. 10), detailed analysis showed that the INT network was more often positioned close to LANG regions than to SAL regions in the replication cohort (*study 3*). Across subjects, INT regions typically extensively bordered the LANG network, were located next to LANG regions in all subjects, even those showing complex anatomy (e.g., see S10 and S12), and were not consistently juxtaposed with SAL regions to the same extent (e.g., see S8).

To test whether the INT network could be defined in additional subjects, and whether the observed spatial relationships with other networks could be replicated, we defined the INT, MOT, and AUD networks in the replication cohort (*study 3*). Figure 14 shows the seed-based estimates of these networks. The key features suggestive of the INT network (described in *The language network abuts an intermediate network that is adjacent to tongue motor and auditory regions*) were replicated in this cohort (*study 3*), but with similar levels of separation from neighboring systems as in the original cohort (*studies 1* and *2*; see Fig. 9). One prominent exception was *subject S11*, where the best estimate of the INT network appeared to show high correlation with regions appearing to be within the dorsal attention network (Corbetta and Shulman 2002) but also included regions circling the AUD network, which led us to believe that the INT network was present. In this individual, the MOT network could also not be separated from dorsal attention network regions to the same extent as in other subjects. In each subject, the sequence of LANG-INT-AUD could be observed in the temporal cortex (Fig. 14), and the LANG-INT-MOT sequence could be observed in pMFG (Figs. 14 and 15).

The relationship between INT and SAL networks was also visualized in the replication cohort. Replicating the original findings, in some subjects, the INT network did not border the SAL network near the pMFG region (see S8 in Fig. 15). In many cases, the INT region in pMFG extensively bordered the LANG network and only partly with SAL regions (see S9, S10, S11, and S13 in Fig. 15). In one instance, despite SAL and LANG containing closely intertwined regions near pMFG, the INT region was situated precisely beside the LANG region (see S12 in Fig. 15). We again did not observe a single case where the INT network bordered the SAL network in pMFG but not the LANG network.

### Study 4

#### Definition of the language network replicates across a third set of individuals.

**Fig. 16.**
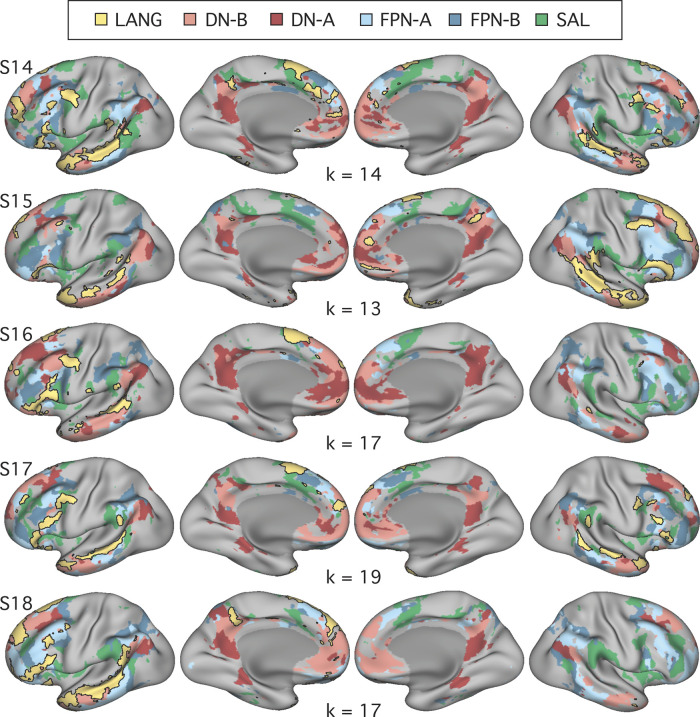
Triplication of close juxtaposition of the language network with neighboring distributed networks revealed by data-driven parcellation. *K*-means clustering was used to parcellate the cortex into *k* networks in each individual from the triplication cohort (*study 4*). Confirming the results from the original (*studies 1* and *2*; see Fig. 4) and replication (*study 3*; see Fig. 11) cohorts, the candidate language network (LANG; yellow and black outline) was observed in all participants (S14–S18). When all subjects were considered, network regions were recapitulated in all of the 9 zones highlighted in Fig. 1. Unusually, subject S15 contained visibly larger regions on the right hemisphere compared with the left. From the parcellation solutions, 5 additional networks were selected for further analysis: the salience network (SAL; green), frontoparietal control network-A and -B (FPN-A and FPN-B; blue), and default network-A and -B (DN-A and DN-B; red).

Five subjects (S14–S18) were analyzed as part of a prospective triplication of the findings from *studies 1*, *2* and *3*. Network definition procedures were repeated from *study 3* without changes. A candidate language network could be defined in all five subjects using both seed-based (Fig. 19) and data-driven clustering approaches (Fig. 16). In all subjects, the LANG network contained prominent regions in the left pMFG, IFG, pSTC, and pSFG. Regions could also be seen in all subjects in the TP and aSFG. Of the remaining nine zones described in the initial experiments (see Fig. 1), four subjects showed a region in MCC (S14, S16, S17, and S18; Fig. 16), three subjects showed a region in dPMC (S14, S15, and S18; Fig. 16), and three subjects showed a region in aITC (S14, S15, and S18; Fig. 16). Three subjects showed a region in the putative ventromedial prefrontal cortex zone (S14, S16, and S18). In the seed-based connectivity maps (lateral view shown on Fig. 19; medial view not shown), additional hints of smaller regions were observed in the aITC (S15 and S16), dPMC (S16), and ventromedial prefrontal cortex (S15).

Two subjects (S16 and S18; Fig. 16) did not show right-hemisphere homologs of the more prominent left-hemisphere regions. Visual inspection of the seed-based maps for these subjects revealed hints of small right-hemisphere regions. One subject, S15, showed an inverted pattern, with clear and more prominent LANG network regions on the right hemisphere and smaller homologous regions on the left hemisphere.

The juxtaposition of DN-B and LANG networks was replicated in *study 4* (Fig. 16), with their interdigitation along IFG present in three subjects (S15, S16, and S17). The sequence of LANG-DN-B-DN-A was evident in the parietal lobe in four individuals (S14, S15, S16, and S18). DN-A regions bordered LANG regions in some subjects in the IFG (S16) and lateral temporal cortex (S14, S16, and S17). Juxtaposition of LANG and FPN-B was again observed in the IFG and pSFG. In the anterior inferior parietal lobe and/or temporoparietal junction, LANG and SAL regions were juxtaposed in four subjects (S14. S16, S17, and S18).

#### Triplication of language network response to language task demands.

**Fig. 17.**
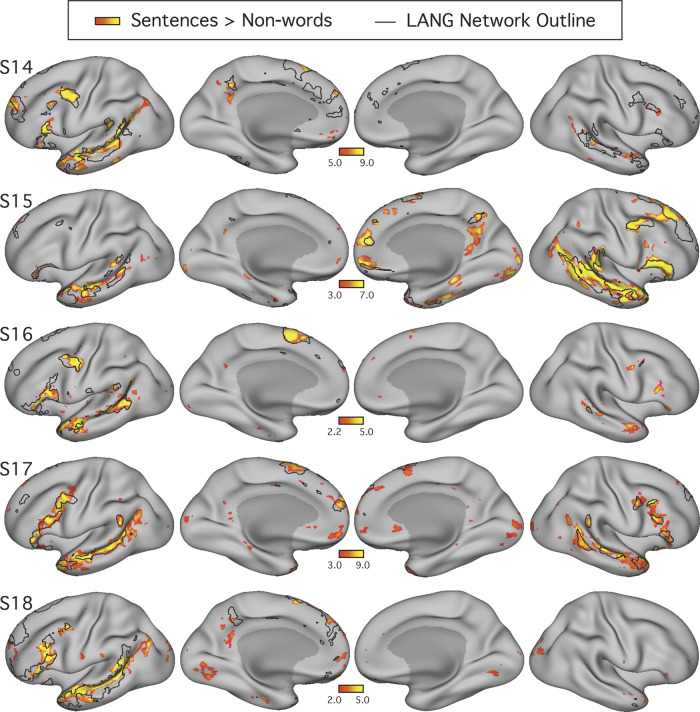
Triplication of close spatial correspondence between the language network and regions activated during a language task contrast. Analysis of the triplication cohort (*study 4*) recapitulated the findings from the original (*studies 1* and *2*; see Fig. 5) and replication (*study 3*; see Fig. 12) cohorts. The language network (LANG) is shown in black outline and was defined using *k*-means clustering (Fig. 16). Red-yellow color bars show within-individual *z*-normalized β-values (i.e., “increased activation”) for the contrast of reading sentences vs. reading lists of nonwords. In all subjects (S14–S18), the language task activations fell predominantly within the boundaries of the intrinsically defined candidate language network. *Subject S15* showed larger regions of activation in the right than in the left hemisphere, corresponding to this subject’s unusual right-lateralized LANG network (see Fig. 16). The detailed anatomy of the distributed intrinsic network corresponded closely with regions showing task-driven activation, including in smaller areas extending beyond the classical language zones (e.g., see S14 and S17), again suggesting that the entire intrinsically organized network is functionally specialized.

Figure 17 shows the boundaries of the LANG network overlaid onto the activation map for the task contrast of reading sentences > nonword lists. The main findings were broadly replicated. The correspondence between connectivity-based and task activation maps can readily be seen in a majority of subjects (S14, S16, and S17; also see close correspondence in right hemisphere of S15 in Fig. 17). Regions showing task activation were largely confined to the boundaries of the intrinsic connectivity network, and in many instances the borders of the LANG network coincided with those of task-activated regions. Evidence for activation within the LANG network was found throughout the distributed network, including smaller regions in dPMC (S14 and S18), aITC (S15, S16, and S18), MCC (S17), and ventromedial prefrontal cortex (S14, S15, S17, and S18) as well as contralateral regions (S17; Fig. 17).

Of special interest is that *subject S15*, who had shown an inverted lateralization pattern with larger LANG network regions on the right hemisphere, also showed much more prominent right-hemisphere regions of activation for the language task contrast. This activation also occurred predominantly within the boundaries of the network as defined by functional connectivity, including alignment of boundaries and evidence for task activation throughout the distributed network in the right hemisphere.

Some of the exceptions noted in the original cohort were also found here. A region of activation was observed in the angular gyrus in *subjects S17* and *S18* and perhaps S14. Hints of the DN-A network were also observed in two individuals (S15 and S17).

**Fig. 18.**
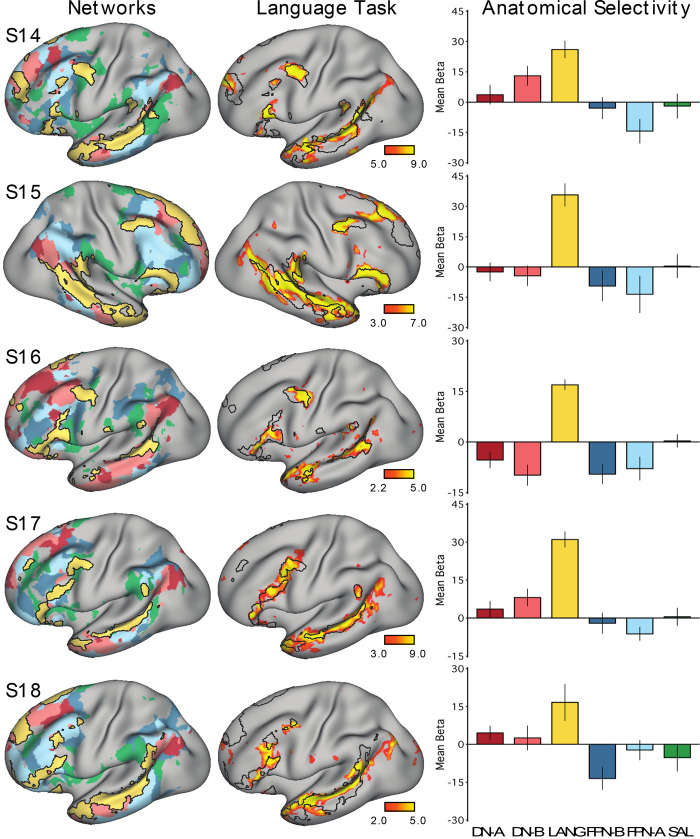
Triplication of selective activation of the language network during a language task contrast. Analysis of the triplication cohort (*study 4*) recapitulated the findings from the original (*studies 1* and *2*; see Fig. 6) and replication (*study 3*; see Fig. 13) cohorts. *Left*: the networks defined by intrinsic functional connectivity from Fig. 16 are replotted. The candidate language network (LANG) is shown in yellow, with the salience network (SAL) in green, the frontoparietal control networks (FPN-A and FPN-B) in blue, and the default networks (DN-A and DN-B) in red. *Middle*: task activation for the contrast of reading sentences vs. reading lists of nonwords are replotted from Fig. 17. *Right*: bar graphs show the mean β-values for the sentences > nonwords contrast, averaged within each within-individual a priori-defined network, along with the standard error of the mean. LANG was the only network showing consistently higher activation for sentences > nonwords, showed the highest mean activation of all the networks in all subjects, and in most cases (S15, S16 and S18) was the only network that showed activity clearly above baseline in the task contrast.

The average language task activation effect (mean β-value) was calculated within the a priori-defined networks for the *study 4* cohort (Fig. 18). This analysis again found selectivity for language task responses within the LANG network. In all subjects, the LANG network showed the highest average task response and considerably higher activation than other networks. In two subjects (S15 and S16), the LANG network was the only network showing activation above baseline (see also S18). In *subjects S14* and *S17*, DN-B showed some evidence of task engagement. With the exception of DN-B in these two subjects, the other networks showed limited if any evidence of task recruitment.

#### Replication of observation of intermediate network across third set of individuals.

**Fig. 19.**
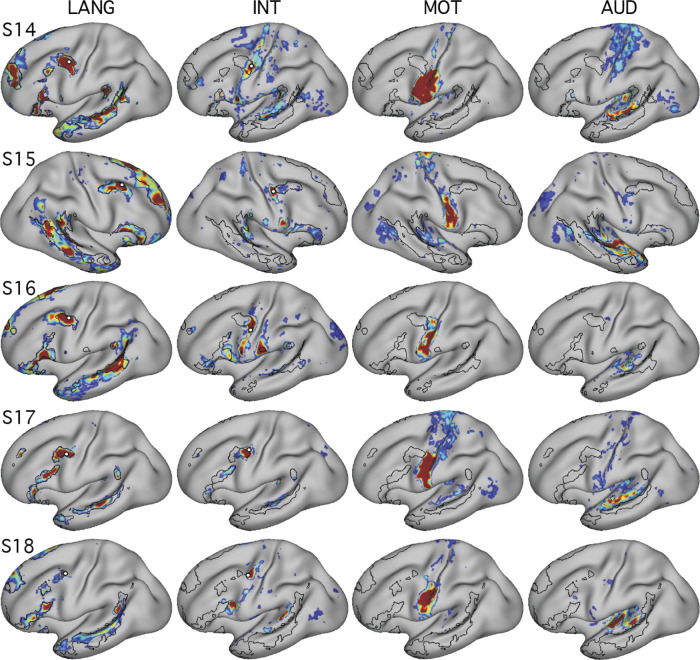
Triplication of distributed networks linking language regions with tongue motor and auditory regions. Generalizing the findings from *studies 1* and *2* (Figs. 7, 8, and 9) and *study 3* (see Fig. 14), intrinsic seed-based connectivity was used to confirm the presence of language (LANG), intermediate (INT), motor (MOT), and auditory (AUD) networks in the triplication cohort (*study 4*; S14–S18). Black outlines display the parcellation-defined intrinsic language network (Fig. 16). White-filled circles denote the location of the seed used to define correlation patterns in that panel. Dashed circles refer to the reflected location of contralateral seed locations. A network that included regions following the expected distribution of the INT network could be defined in all subjects.

**Fig. 20.**
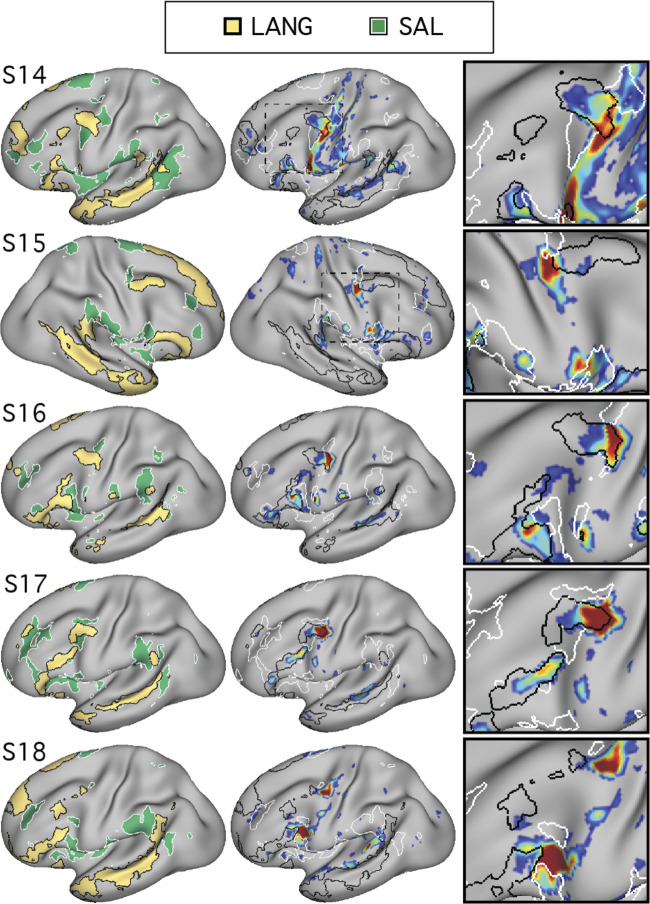
Triplication of spatial relationships between the intermediate (INT), language (LANG), and salience (SAL) networks. Replicating the results from the original (*studies 1* and *2*; see Fig. 10) and replication cohorts (*study 3*; see Fig. 15), detailed analysis showed that the INT network was more often positioned next to the LANG regions than the SAL regions in the triplication cohort (*study 4*). Across subjects, INT regions typically extensively bordered the LANG network, were located next to LANG regions in all subjects, and were not consistently juxtaposed with SAL regions to the same extent (e.g., see S16 and S17).

Figure 19 shows the seed-based estimates of the INT, MOT, and AUD networks for the triplication cohort (*study 4*). The key features suggestive of the INT network (described in *The language network abuts an intermediate network that is adjacent to tongue motor and auditory regions*) were observed in each of the individuals in the triplication cohort. The sequence of LANG-INT-AUD regions could be observed in the temporal cortex (Fig. 19), and the LANG-INT-MOT sequence could be observed in pMFG (Figs. 19 and 20). Note that for *subject S15* the right hemisphere was analyzed (using left hemisphere seed for MOT and AUD networks) given this subject’s clear right-lateralized LANG network anatomy. In S15, the network sequences could be defined in both hemispheres. In *subject S16*, the best estimate of the INT network also showed correlation with regions in or near the inferior postcentral and precentral gyri. However, this map also included regions characteristic of INT, such as the discrete regions surrounding AUD regions and situated next to LANG regions in lateral temporal cortex. In *subjects S14* and *S15*, the seed selected for the INT network produced noisy maps with diffuse regions of low correlation. In these maps, the higher correlation (red) vertices in regions of the INT network helped build confidence that the network was being defined (Fig. 19).

The relationship between INT and SAL networks was also visualized in the triplication cohort (Fig. 20). Previous observations were again broadly replicated. In a majority of cases, the INT region in pMFG extensively bordered the LANG network and only partly with SAL network regions (see subjects S14, S16 and S17 in Fig. 20). We also did not observe a single case where the INT network borders the SAL network in pMFG but not the LANG network.

#### Composite analyses.

**Fig. 21.**
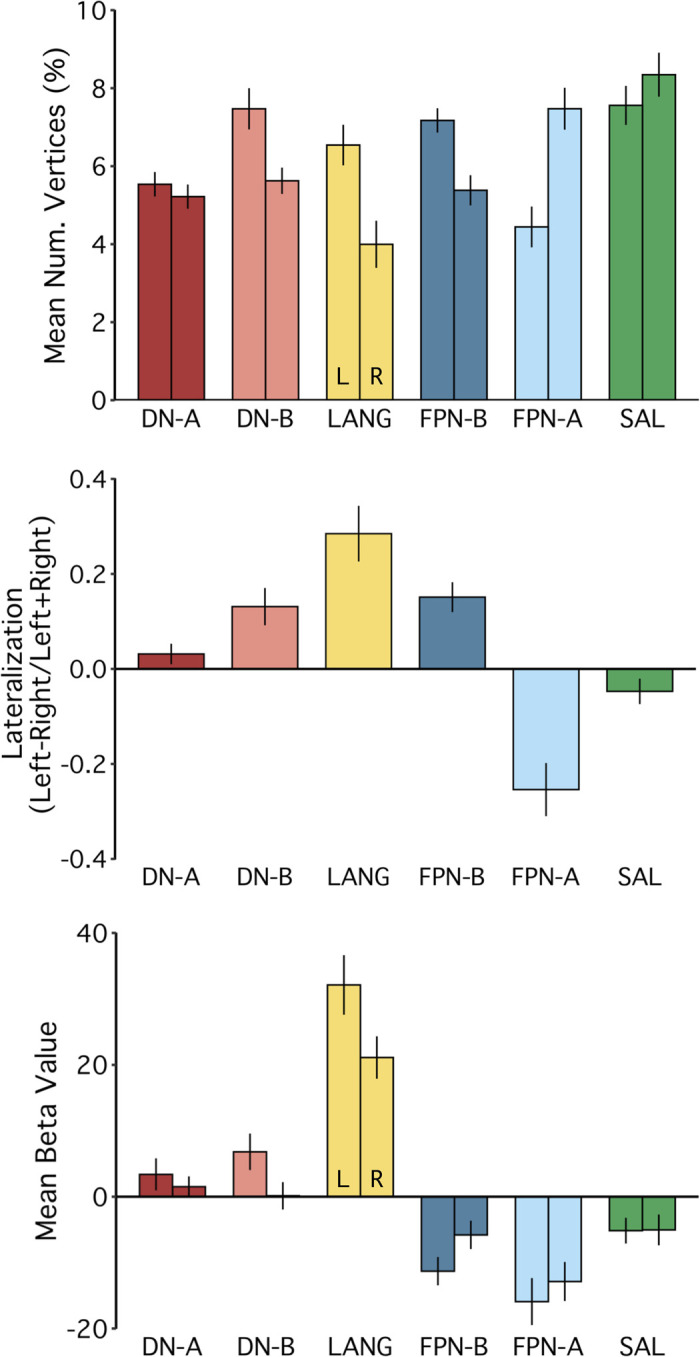
The language network is left lateralized on average in the group. Composite analyses were conducted using all 18 subjects from the original, replication, and triplication cohorts (*studies 1–4*). Group means are plotted in each panel, with standard error of the mean. *Top*: %total vertices in the left (L) and right (R) hemispheres included in each of the 6 a priori networks was calculated as a proxy for the relative size or surface area occupied by each network. The language network (LANG) showed larger regions on the left compared with right hemispheres, as did default network B (DN-B) and frontoparietal control network B (FPN-B). Default network A (DN-A) and the salience network (SAL) showed no or limited evidence of consistent lateralization. Frontoparietal control network A (FPN-A) showed a consistent right-lateralized pattern. *Middle*: direct comparison of the relative size of network regions in each hemisphere revealed that, on average, the LANG network was the most left lateralized of the networks tested. The lateralization metric computed was the number of network vertices in the left hemisphere minus the number of network vertices in the right divided by the total number of network vertices in both hemispheres. Positive values denote left lateralization, and negative values denote right lateralization. *Bottom*: the group mean β-value for the contrast of reading sentences vs. lists of pronounceable nonwords was calculated for vertices falling within each a priori network, separated by hemisphere. Despite differences in the relative size of LANG network regions in each hemisphere, robust evidence for activation in both hemispheres was observed, with left hemisphere regions showing higher levels of activity.

Figure 21 shows the results of composite analyses that combined data across all four studies (*n* = 18) to explore network properties. On average, the LANG network was left-lateralized in the sense of containing larger regions on the left hemisphere (Fig. 21, *top*). Note that size was approximated here by the proportion of total vertices in each hemisphere that were contained within each network. Other networks also showed a degree of lateralization, such as DN-B and FPN-B, which showed evidence of left lateralization, and FPN-A, which was strongly right lateralized. The LANG network had the highest difference in region size between left and right hemispheres (Fig. 21, *middle*). That is, by this measure the LANG network was the most left-lateralized out of the six a priori networks studied.

The selectivity of language task responses for each network was quantified for each cohort using the full bilateral estimates of the networks (Figs. 6, 13, and 18). However, one possibility is that this selectivity is confined to one hemisphere or that within the LANG network one hemisphere is preferentially activated. Figure 21, *bottom*, shows the mean β-value for the language task contrast taken from each network, split by hemisphere. Both left and right hemisphere portions of the LANG network showed clear increased activation during the contrast of reading sentences > nonword lists. In both hemispheres, the LANG network was activated to a level well above that observed for the other networks. The left hemisphere LANG regions showed higher β-values than those in the right hemisphere. Although this might support that the left hemisphere regions are on average more activated than the right hemisphere regions during the language task used here, an alternative explanation is that, given that the right hemisphere contained smaller LANG regions in the majority of subjects, the lower mean β-values in the right hemisphere could be due to a greater influence of partial volume effects. It is difficult to rule out this explanation given the resolution of the present data set. The similar level of task recruitment observed in each hemisphere (Fig. 21, *bottom*) suggests that it is not implausible that LANG network regions are recruited to a similar degree in both hemispheres. A similar pattern of both hemispheres showing increased activity but with left hemisphere regions showing slightly higher activation was consistently observed, in most subjects, when each subject was plotted individually (not shown). Interestingly, for *subject S15*, who had larger regions on the right, the task activation asymmetry was also flipped, with higher activation on the right than on the left.

**Fig. 22.**
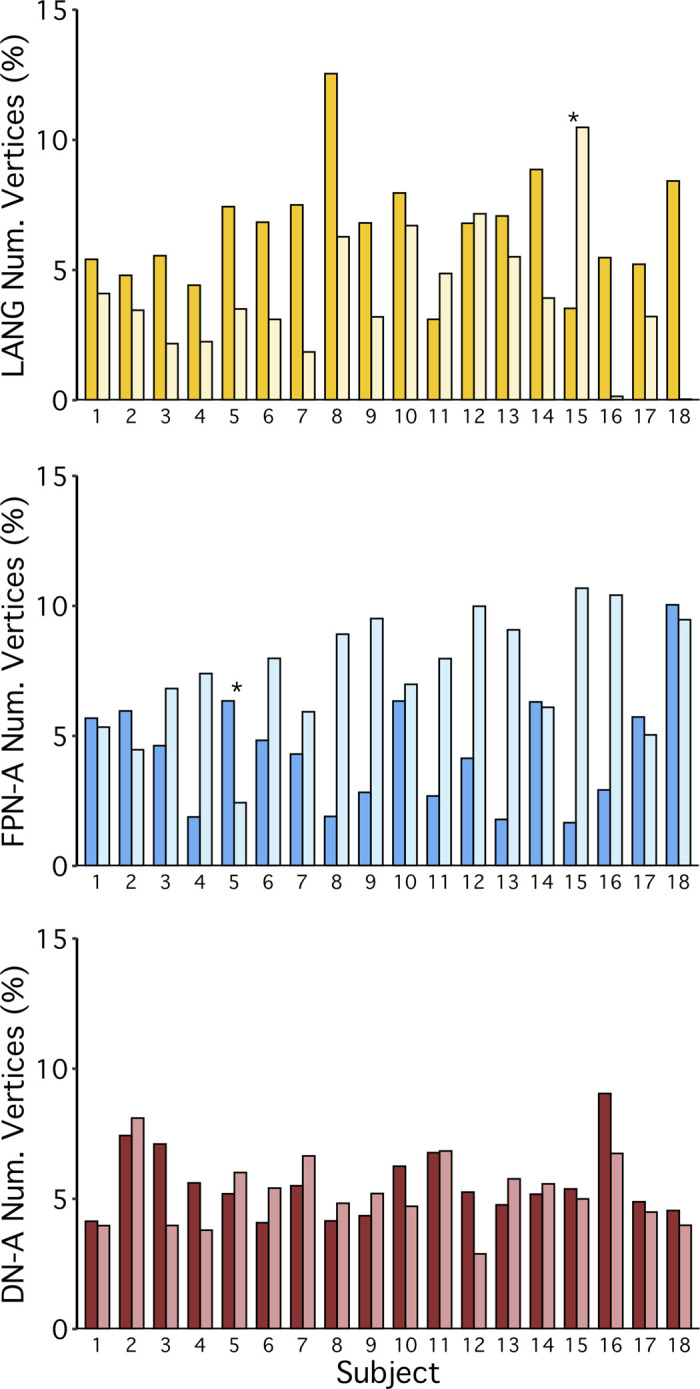
The language network is left lateralized in a majority of individuals, with notable exceptions. Extending the composite analyses conducted using all 18 subjects from the original, replication, and triplication cohorts (*studies 1–4*; Fig. 21), the %total network vertices in the left and right hemispheres was plotted for each subject. The darker-shaded bars represent the left hemisphere, and the lighter-shaded bars represent the right hemisphere. Graphs show the %vertices from each hemisphere contained within the language (LANG; *top*), frontoparietal control network A (FPN-A; *middle*), and default network A (DN-A; *bottom*). The LANG network was left lateralized in 15 out of the total 18 subjects. Of the other networks, FPN-A showed a strong and consistent right-lateralized pattern, and DN-A showed a consistent bilateral pattern. Note that the bilateral pattern for DN-A observed in the group average (see Fig. 21) is consistent across individuals and not a result of mixed strong left and right lateralization in different subjects. *Interesting subjects that showed clear evidence for opposite patterns from the group norm. *Subject S15*, by all analyses (see text), displays a pattern of flipped lateralization of the language network.

A final analysis quantified the degree of lateralization in each subject. Figure 22 shows that, in the great majority of subjects, the LANG network occupied a consistently larger percentage of the total vertices in the left hemisphere than in the right hemisphere. In a few cases (S16 and S18), the extent of lateralization was extreme (although note that in S18 very small right hemisphere regions were observed in a seed-based estimate of the LANG network that were lost in the clustering solution used for the composite analyses). A noteworthy exception (see asterisk in Fig. 22, *top*) was *subject S15*, whose LANG network estimate was clearly right-lateralized. Out of the 18 subjects tested, this was the exception that proved the rule; although this subject was unusual in having a right-lateralized LANG network, their task activation pattern was also right-lateralized, confirming the correspondence between networks as defined by functional connectivity and task activation patterns. The percentage of vertices per hemisphere was also quantified for two other networks: FPN-A, which showed largely consistent right lateralization (Fig. 22, *middle*; and note the most marked exception in *subject S5*, denoted by asterisk), and DN-A, which did not show strong and consistent lateralization toward either hemisphere (Fig. 22, *bottom*).

## DISCUSSION

The present results demonstrate that a distributed language network can be defined within individuals using intrinsic functional connectivity. Organizational details suggest that the network *1*) is distinct but spatially adjacent to the default and frontoparietal control networks throughout the cortex; *2*) has a distributed spatial motif that parallels other association networks; *3*) involves upwards of nine cortical regions in the left hemisphere alone, some of which extend beyond the classical language zones and have not been previously emphasized; and *4*) responds in an anatomically specific manner to language task demands, with adjacent networks showing minimal or no response. We also observed a smaller, distinct distributed network that occupies regions in between the language network and the orofacial motor regions in the frontal lobe and the auditory reception regions in the temporal lobe, suggesting a network hierarchy linking language to functionally related sensorimotor regions. We discuss the implications of these collective observations for understanding the relationship of the language network with the multiple parallel networks that populate association cortex.

### The Language Network Can Be Resolved Within Individuals Using Functional Connectivity

A distributed network that contains regions in classic perisylvian language areas was observed in all 18 individuals tested using intrinsic functional connectivity (Figs. 1, 14, and 19; see also Glasser et al. 2016; Hacker et al. 2013; Hampson et al. 2002; Lee et al. 2012). The network was confirmed across analysis methods (Figs. 1, 2, and 4) and independent data sets within the same individual (Fig. 3) and could be detected by initiating network definition from multiple distributed locations (Fig. 2). The language network occupied regions that were juxtaposed with other association networks, such as the default, frontoparietal control, and salience networks (Fig. 4). The close spatial relationship between neighboring networks, some of which were finely interdigitated (e.g., see sequential LANG and DN-B network regions along the left IFG in Fig. 4), suggests why some prior studies of functional connectivity, particularly those involving data-driven methods and group-averaged data, may have failed to separate the language network from nearby systems like the default network (e.g., see Power et al. 2011; Yeo et al. 2011; but also see Blank et al. 2014 and Mineroff et al. 2018) and also why studies capturing the network may miss its functional significance.

### The Language Network Parallels the Organizational Motif of Other Association Networks

An intriguing observation of the present study is that the language network is just one of multiple similarly organized distributed association networks. The literature has most often focused on specialization of language regions without consideration of how language networks are similar or dissimilar from other distributed association networks. Our results are consistent with a specialized left-lateralized network but also illustrate that the distinct language network is just one of several association networks that share a common organizational motif consisting of widely distributed regions located in multiple cortical zones.

Specifically, the network included classical language regions in the frontal and temporal cortices (IFG, pSTC, pSFG, TP, pMFG) as predicted by clinical and task activation studies (see introduction). However, the network also extended beyond the classical language areas (see Hacker et al. 2013 and Lee et al. 2012). Regions were observed in the parietal (dPMC and possibly pSTC region), midcingulate (MCC), and inferior temporal (aITC) cortices, with a further candidate region within the ventromedial prefrontal cortex (Figs. 1, 4, 11, and 16). An anterior prefrontal region (aSFG) that appeared to be distinct from the pSFG region was also detected. Further regions were detected in the right hemisphere, and these regions again displayed a distributed organization that was in many ways homologous to the spatial distribution observed in the left hemisphere (Fig. 4).

When considered together, the candidate language network parallels the distributed motif characteristic of association cortex in the nonhuman primate (see the 4th figure in Goldman-Rakic 1988; Buckner and Margulies 2019; Margulies et al. 2009; also see Frey et al. 2014; Ghahremani et al. 2017) and previously observed across multiple association networks in humans (Braga and Buckner 2017; Margulies et al. 2016; Power et al. 2011; Yeo et al. 2011). Consistent with earlier observations focused on frontal cortex (Fedorenko et al. 2012), the language network contained side-by-side regions with other well-characterized networks such as the default network, which sits at the apex of a sensory-to-transmodal cortex hierarchy (Buckner and DiNicola 2019; Buckner and Margulies 2019; Margulies et al. 2016). Neighboring language and DN-B network regions were observed in multiple cortical zones (Figs. 4, 11, and 16). The present characterization further illustrates that the spatial juxtapositions are present for multiple distributed components of the language network across the cortex.

For example, the default network contains distributed regions along the posterior, middle, and anterior cortical midline, including at or near the posterior cingulate, and along the frontal midline (see zones 5–9 in the 3rd figure in Braga and Buckner 2017 and detailed anatomy in Braga et al. 2019b). The language network regions were observed within each of these zones (Fig. 1), with regions reliably identified within the posterior (dPMC; zone 5 in Braga and Buckner 2017), middle (MCC; zone 6), and anterior cortical midline at the pSFG (zone 7), aSFG (zone 8), and potentially ventromedial prefrontal cortex (zone 9). Along the lateral surface, language regions were also observed near or directly bordering DN-B in the four remaining zones highlighted in the 3rd figure of Braga and Buckner 2017, including the IFG, aSFG and pSFG, TP, and pSTC. The posterior parietal pSTC region also bordered the prominent default network regions in the inferior parietal lobule (Figs. 4, 11, and 16).

The side-by-side relationship between the language network and other distributed association networks could only fully be appreciated when the smaller midline regions were resolved within individuals. This reinforces the notion that the association cortices are organized into parallel distributed networks and that in this sense the language network is a characteristic association network.

### Task Activation Is Selective for the Language Network

By collecting data during a language localizer task performed by each of our volunteers, we were able to test the hypothesis that the language network, as defined by intrinsic connectivity, is activated by language task demands and also explore the anatomic specificity of the response (see also Glasser et al. 2016). Overlap between connectivity and task activation maps was observed throughout the cortical mantle (Figs. 5, 12, and 17). In many cases, the idiosyncratic shape of language network regions closely matched task-activated patterns (e.g., see S1, S2, S5, and S6 in Fig. 5) despite being defined in independent data and based on different analysis principles. Importantly, this correspondence extended beyond the classical language regions and often included the smaller regions of the language network (see Figs. 5, 12, and 17). Notable examples include the dPMC region in S2, S4, S5, S7, S8, S10, S14, and S18, the aSFG region in S1, S2, S3, S4, S5, S6, S7, S8, S9, S11, S14, and S17, the aITC region in S1, S4, S5, S6, S9, S15, and S18, and even the ventromedial prefrontal cortex region in S1, S5, S7, S10, and S14. The small MCC region showed evidence of task activation in S5 and S17 at the thresholds selected.

The finding of task activation in these smaller midline regions suggests that, under certain task conditions, the entire distributed network is recruited simultaneously in a coordinated manner. In other words, the domain-specialized module appears to be the distributed network, not simply localized regions (see also DiNicola et al. 2020).

The alignment between task activation and connectivity was not perfect, and there was variability across subjects in how well the two maps aligned. Possible explanations for a discrepancy in some individuals include *1*) that some subjects may have deployed alternative strategies during either of the language task conditions or found one condition easier than the other, *2*) differences in the extent to which the clustering analysis was able to separate the LANG network from neighboring networks in some individuals, and *3*) errors in the manual identification of networks. The fact that there were multiple individuals in which a close correspondence was clear (see especially S2, S3, S4, S5, S6, and S7 in Fig. 5) and that this finding replicated (see especially S11 and S13 in Fig. 12) and triplicated (see especially S14, S16, and S17 in Fig. 17) builds support for the idea that the entire distributed network is specialized for linguistic processes. Furthermore, the findings support that the distributed network is functionally specialized in ways that are distinct from spatially adjacent networks.

The correspondence between functional connectivity and task activation has been noted before (e.g., see Buckner et al. 2008; Glasser et al. 2016; Gordon et al. 2017b; Ji et al. 2019; Smith et al. 2009). Recently, Tavor et al. (2016) showed that functional connectivity can predict idiosyncratic task activation patterns across individuals. Glasser et al. (2016) also showed that language activation patterns can be recapitulated by intrinsic connectivity using seeds placed in the pMFG and pSTC (see also Hampson et al. 2002). Here, we provide additional evidence for spatial specificity. When the average task activation effect was calculated for six distributed networks identified a priori, the language network showed robust and selective response during the language task (Figs. 6, 13, and 18). This was despite the fact that the other networks often possessed regions closely positioned near the language network simultaneously in multiple cortical zones.

Further support comes from the observation of a minority of individuals (S11, S12, and S15) who did not display a left-lateralized LANG network. In contrast to most subjects, S15 was strongly right lateralized, a pattern that was observed in only 1 out of 18 (∼6%) of the individuals tested here. This exception confirmed two observations. First, the LANG network contains homologous regions in both hemispheres. In *subject S15*, the LANG network was initially identified based on the distribution of regions on the left hemisphere, which occupied the expected zones identified in Fig. 1, but with smaller regions than expected, particularly along lateral and medial frontal cortices. The observation of more prominent regions in the right hemisphere was made after the network had been identified. Hence, even in this individual’s “nondominant” hemisphere, the LANG network could be identified based on the distribution of homologous regions.

Second, the LANG network overlaps with regions showing increased task activation for the language contrast in a manner that respects each individual’s idiosyncratic functional anatomy. In contrast to most subjects, the language localizer task activation map for *subject S15* was also strongly right lateralized, with larger regions of increased activation on the right hemisphere. Despite this subject having an unusually right-lateralized LANG network, the correspondence with task activation remained, with both maps showing clear overlap and similar boundaries, including larger regions on the right (Fig. 17) and increases in task-driven activity that were predominantly within the bounds of the LANG network as defined by connectivity (Fig. 18).

The other two exceptions were *subjects S11* and *S12*. When the relative size of LANG network regions was compared between left and right hemispheres, these two subjects showed a bilateral if weakly right-lateralized pattern (Fig. 22). In these two subjects, the task activation maps also showed larger regions on the right hemisphere than on the left (Fig. 12), with vertices showing increased activation again being located largely within the bounds of the LANG network. This was not observed for any of the other subjects, all of which had larger task-driven responses on the left than on the right. The unusual network patterns observed in S11, S12, and S15 provide evidence for a relationship between connectivity and task activation, further supporting that the topography of the intrinsically connected network may arise from a process of network differentiation and functional specialization for linguistic processes.

### An Intermediate Network Abuts the Language Network as well as Orofacial Motor and Auditory Regions

Motivated by the hypothesis that the location of prominent language network regions may be explained by their proximity to orofacial motor and auditory regions, we initially explored the functional anatomy of these regions in two individuals (Figs. 7 and 8). Rather than being juxtaposed, we unexpectedly found a slight separation between sensorimotor regions and the language network in both frontal and temporal cortices. When the functional anatomy of this gap was explored using a seed-based approach, we observed a distinct “intermediate” (INT) network that had a distributed organization and occupied neighboring cortical regions to the LANG network in both lateral frontal and temporal cortices (Figs. 7 and 8) as well as along the dorsal posterior frontal midline (not shown). In the frontal lobe, the INT network bordered the LANG network at both dorsolateral (pMFG), dorsomedial (pSFG), and ventrolateral (IFG) locations. In these two individuals, a motor task allowed us to functionally define inferior sensorimotor regions. The motor task included only tongue movements, and it was not possible to map out motor regions involved in the movement of other articulators (lips, pharynx) or the vocal folds (Brown et al. 2008; Carey et al. 2017; Hesselmann et al. 2004; see also Petrides et al. 2005). Hesselmann et al. (2004) previously showed that lip movements activate a motor region more dorsal than ventral motor regions activated for tongue movements. One might speculate that the pMFG and IFG INT network regions are associated with different laropharyngeal movements related to independent aspects of articulation and vocalization (see Fig. 3 in de Heer et al. 2017).

The presence of the INT network was confirmed to some degree in all 18 individuals across the three cohorts (Figs. 9, 14, and 19). Although the network was separated from nearby networks to varying degrees of success, the key features of the INT network (juxtaposition with LANG regions in pMFG, IFG, pSFG, and pSTC) were reliably observed in all individuals. In a majority of the individuals (*n* = 10; S1, S2, S6, S8, S10, S12–15, and S18; see Figs. 9, 14, and 19), we were able to detect the referential INT features in a network that was clearly separate from other nearby networks; i.e., the INT network occupied distinct regions of the cortex. In the remaining eight individuals, our best estimate of the INT network overlapped with other networks but still contained the referential features. These inconsistencies may be due to the small size of the INT network and close juxtaposition with other functional zones, which we believe puts the INT network close to the resolution limits of the present data. Future studies with higher resolution will be needed to confirm or refute the hypothesis of a functionally separate INT network.

The spatial relationships observed also raise the possibility that the LANG and INT networks form a sequence of functional regions that is repeated in multiple cortical zones. The sequence links language regions with tongue movement regions (LANG-INT-MOT) in pMFG (Figs. 7*B* and 8*B*) and with auditory regions (LANG-INT-AUD) in the temporal lobe (Figs. 7*C* and 8*C*). The result is a parallel sequence of distributed networks that fall along a gradient from language regions to sensorimotor and possibly other association networks (also see Braga and Buckner 2017; Buckner and Margulies 2019; Margulies et al. 2016; Power et al. 2011).

Following the sequence into transmodal cortex, the LANG network also displayed regions neighboring DN-B in many cortical zones (Fig. 4). In particular, DN-B contains a region in anterior IFG that is closely interdigitated with the LANG network region and extends the sequence into anterior IFG (i.e., DN-B-LANG-INT-MOT). Similarly, a DN-B region is found in the inferior parietal cortex at or near the temporoparietal junction, which also can be seen as an extension of the sequence into the parietal lobe (i.e., DN-B-LANG–INT-AUD). Altogether, these observations situate the LANG network as falling along a gradient of distributed networks that link auditory and motor cortices with transmodal cortices that support higher-level cognitive functions.

### Conclusions

The present study extends our understanding of the language network by showing that the distributed organization of the language network parallels that of other association networks. We reveal the close spatial relationships between language network regions and other distributed systems in classic language regions and show that the language network sits within a large-scale gradient linking sensorimotor and higher-level association networks. We also resolve small language regions in both hemispheres that have not previously been emphasized and show that these are also language responsive. The close correspondence of the language network defined by functional connectivity and task activation suggests that precision functional mapping could aid applied endeavors targeting the language network, such as intracranial neuromodulation, or limit complications from surgical resection. Such an approach might be particularly useful for clinical populations that may be unable to perform tasks in the scanner.

## GRANTS

R.M.B. was supported by Wellcome Trust Grant 103980/Z/14/Z and National Institutes of Health (NIH) Pathway to Independence Award Grant K99MH117226. L.M.D. was supported by National Science Foundation Grant DGE-1745303 (the opinions, findings, and conclusions expressed in this material are those of the authors and do not necessarily reflect the views of the National Science Foundation). This work was also supported by Kent and Liz Dauten, NIH Grant P50-MH-106435, and Shared Instrumentation Grant S10OD020039.

## DISCLOSURES

No conflicts of interest, financial or otherwise, are declared by the authors.

## AUTHOR CONTRIBUTIONS

R.M.B. and R.L.B. conceived and designed research; R.M.B., L.M.D., H.C.B., and R.L.B. performed experiments; R.M.B., L.M.D., H.C.B., and R.L.B. analyzed data; R.M.B. and R.L.B. interpreted results of experiments; R.M.B., L.M.D., and R.L.B. prepared figures; R.M.B., L.M.D., and R.L.B. drafted manuscript; R.M.B., L.M.D., H.C.B., and R.L.B. edited and revised manuscript; R.M.B., L.M.D., H.C.B., and R.L.B. approved final version of manuscript.

## References

[B1] Bates E, Wilson SM, Saygin AP, Dick F, Sereno MI, Knight RT, Dronkers NF. Voxel-based lesion-symptom mapping. Nat Neurosci 6: 448–450, 2003. doi:10.1038/nn1050. 12704393

[B2] Binder JR, Desai RH, Graves WW, Conant LL. Where is the semantic system? A critical review and meta-analysis of 120 functional neuroimaging studies. Cereb Cortex 19: 2767–2796, 2009. doi:10.1093/cercor/bhp055. 19329570 PMC2774390

[B3] Binder JR, Rao SM, Hammeke TA, Frost JA, Bandettini PA, Jesmanowicz A, Hyde JS. Lateralized human brain language systems demonstrated by task subtraction functional magnetic resonance imaging. Arch Neurol 52: 593–601, 1995. doi:10.1001/archneur.1995.00540300067015. 7763208

[B4] Blank I, Kanwisher N, Fedorenko E. A functional dissociation between language and multiple-demand systems revealed in patterns of BOLD signal fluctuations. J Neurophysiol 112: 1105–1118, 2014. doi:10.1152/jn.00884.2013. 24872535 PMC4122731

[B5] Blank SC, Scott SK, Murphy K, Warburton E, Wise RJS. Speech production: Wernicke, Broca and beyond. Brain 125: 1829–1838, 2002. doi:10.1093/brain/awf191. 12135973

[B6] Blumstein SE, Amso D. Dynamic functional organization of language: insights from functional neuroimaging. Perspect Psychol Sci 8: 44–48, 2013. doi:10.1177/1745691612469021. 25414726 PMC4235529

[B7] Braga RM, Buckner RL. Parallel interdigitated distributed networks within the individual estimated by intrinsic functional connectivity. Neuron 95: 457–471.e5, 2017. doi:10.1016/j.neuron.2017.06.038. 28728026 PMC5519493

[B8] Braga RM, DiNicola LM, Buckner RL. Situating the left-lateralized language network in the broader organization of multiple specialized large-scale distributed networks (Preprint) bioRxiv: 1–26, 2019 a. doi:10.1101/2019.12.11.873174. PMC8356783 32965153

[B9] Braga RM, Van Dijk KRA, Polimeni JR, Eldaief MC, Buckner RL. Parallel distributed networks resolved at high resolution reveal close juxtaposition of distinct regions. J Neurophysiol 121: 1513–1534, 2019 b. doi:10.1152/jn.00808.2018. 30785825 PMC6485740

[B10] Braze D, Mencl WE, Tabor W, Pugh KR, Constable RT, Fulbright RK, Magnuson JS, Van Dyke JA, Shankweiler DP. Unification of sentence processing via ear and eye: an fMRI study. Cortex 47: 416–431, 2011. doi:10.1016/j.cortex.2009.11.005. 20117764 PMC2889140

[B11] Brown S, Ngan E, Liotti M. A larynx area in the human motor cortex. Cereb Cortex 18: 837–845, 2008. doi:10.1093/cercor/bhm131. 17652461

[B12] Buckner RL, Andrews-Hanna JR, Schacter DL. The brain’s default network: anatomy, function, and relevance to disease. Ann N Y Acad Sci 1124: 1–38, 2008. doi:10.1196/annals.1440.011. 18400922

[B13] Buckner RL, DiNicola LM. The brain’s default network: updated anatomy, physiology and evolving insights. Nat Rev Neurosci 20: 593–608, 2019. doi:10.1038/s41583-019-0212-7. 31492945

[B14] Buckner RL, Krienen FM, Castellanos A, Diaz JC, Yeo BTT. The organization of the human cerebellum estimated by intrinsic functional connectivity. J Neurophysiol 106: 2322–2345, 2011. doi:10.1152/jn.00339.2011. 21795627 PMC3214121

[B15] Buckner RL, Margulies DS. Macroscale cortical organization and a default-like apex transmodal network in the marmoset monkey. Nat Commun 10: 1976, 2019. doi:10.1038/s41467-019-09812-8. 31036823 PMC6488644

[B16] Burianova H, Grady CL. Common and unique neural activations in autobiographical, episodic, and semantic retrieval. J Cogn Neurosci 19: 1520–1534, 2007. doi:10.1162/jocn.2007.19.9.1520. 17714013

[B17] Carey D, Krishnan S, Callaghan MF, Sereno MI, Dick F. Functional and quantitative MRI mapping of somatomotor representations of human supralaryngeal vocal tract. Cereb Cortex 27: 265–278, 2017. doi:10.1093/cercor/bhw393. 28069761 PMC5808730

[B18] Corbetta M, Shulman GL. Control of goal-directed and stimulus-driven attention in the brain. Nat Rev Neurosci 3: 201–215, 2002. doi:10.1038/nrn755. 11994752

[B19] Cox RW. AFNI: software for analysis and visualization of functional magnetic resonance neuroimages. Comput Biomed Res 29: 162–173, 1996. doi:10.1006/cbmr.1996.0014. 8812068

[B20] Cox RW. AFNI: what a long strange trip it’s been. Neuroimage 62: 743–747, 2012. doi:10.1016/j.neuroimage.2011.08.056. 21889996 PMC3246532

[B21] de Heer WA, Huth AG, Griffiths TL, Gallant JL, Theunissen FE. The hierarchical cortical organization of human speech processing. J Neurosci 37: 6539–6557, 2017. doi:10.1523/JNEUROSCI.3267-16.2017. 28588065 PMC5511884

[B22] DiNicola LM, Braga RM, Buckner RL. Parallel distributed networks dissociate episodic and social functions within the individual. J Neurophysiol 123: 1144–1179, 2020. doi:10.1152/jn.00529.2019. 32049593 PMC7099479

[B23] Dosenbach NUF, Fair DA, Miezin FM, Cohen AL, Wenger KK, Dosenbach RAT, Fox MD, Snyder AZ, Vincent JL, Raichle ME, Schlaggar BL, Petersen SE. Distinct brain networks for adaptive and stable task control in humans. Proc Natl Acad Sci USA 104: 11073–11078, 2007. doi:10.1073/pnas.0704320104. 17576922 PMC1904171

[B24] Doucet G, Naveau M, Petit L, Delcroix N, Zago L, Crivello F, Jobard G, Tzourio-Mazoyer N, Mazoyer B, Mellet E, Joliot M. Brain activity at rest: a multiscale hierarchical functional organization. J Neurophysiol 105: 2753–2763, 2011. doi:10.1152/jn.00895.2010. 21430278

[B25] Duncan J. The multiple-demand (MD) system of the primate brain: mental programs for intelligent behaviour. Trends Cogn Sci 14: 172–179, 2010. doi:10.1016/j.tics.2010.01.004. 20171926

[B26] Fedorenko E, Behr MK, Kanwisher N. Functional specificity for high-level linguistic processing in the human brain. Proc Natl Acad Sci USA 108: 16428–16433, 2011. doi:10.1073/pnas.1112937108. 21885736 PMC3182706

[B27] Fedorenko E, Duncan J, Kanwisher N. Language-selective and domain-general regions lie side by side within Broca’s area. Curr Biol 22: 2059–2062, 2012. doi:10.1016/j.cub.2012.09.011. 23063434 PMC3494832

[B28] Fedorenko E, Hsieh PJ, Nieto-Castañón A, Whitfield-Gabrieli S, Kanwisher N. New method for fMRI investigations of language: defining ROIs functionally in individual subjects. J Neurophysiol 104: 1177–1194, 2010. doi:10.1152/jn.00032.2010. 20410363 PMC2934923

[B29] Ferstl EC, Neumann J, Bogler C, von Cramon DY. The extended language network: a meta-analysis of neuroimaging studies on text comprehension. Hum Brain Mapp 29: 581–593, 2008. doi:10.1002/hbm.20422. 17557297 PMC2878642

[B30] Fischl B, Sereno MI, Dale AM. Cortical surface-based analysis. II: Inflation, flattening, and a surface-based coordinate system. Neuroimage 9: 195–207, 1999. doi:10.1006/nimg.1998.0396. 9931269

[B30a] Frey S, Mackey S, Petrides M. Cortico-cortical connections of areas 44 and 45B in the macaque monkey. Brain Lang 131: 36–55, 2014. doi:10.1016/j.bandl.2013.05.005. 24182840

[B31] Geschwind N. The organization of language and the brain. Science 170: 940–944, 1970. doi:10.1126/science.170.3961.940. 5475022

[B32] Ghahremani M, Hutchison RM, Menon RS, Everling S. Frontoparietal functional connectivity in the common marmoset. Cereb Cortex 27: 3890–3905, 2017. doi:10.1093/cercor/bhw198. 27405331

[B33] Glasser MF, Coalson TS, Robinson EC, Hacker CD, Harwell J, Yacoub E, Ugurbil K, Andersson J, Beckmann CF, Jenkinson M, Smith SM, Van Essen DC. A multi-modal parcellation of human cerebral cortex. Nature 536: 171–178, 2016. doi:10.1038/nature18933. 27437579 PMC4990127

[B34] Gold BT, Buckner RL. Common prefrontal regions coactivate with dissociable posterior regions during controlled semantic and phonological tasks. Neuron 35: 803–812, 2002. doi:10.1016/S0896-6273(02)00800-0. 12194878

[B35] Goldman-Rakic PS. Topography of cognition: parallel distributed networks in primate association cortex. Annu Rev Neurosci 11: 137–156, 1988. doi:10.1146/annurev.ne.11.030188.001033. 3284439

[B36] Gordon EM, Laumann TO, Adeyemo B, Petersen SE. Individual variability of the system-level organization of the human brain. Cereb Cortex 27: 386–399, 2017 a. doi:10.1093/cercor/bhv239. 26464473 PMC5939195

[B37] Gordon EM, Laumann TO, Gilmore AW, Newbold DJ, Greene DJ, Berg JJ, Ortega M, Hoyt-Drazen C, Gratton C, Sun H, Hampton JM, Coalson RS, Nguyen AL, McDermott KB, Shimony JS, Snyder AZ, Schlaggar BL, Petersen SE, Nelson SM, Dosenbach NUF. Precision functional mapping of individual human brains. Neuron 95: 791–807.e7, 2017 b. doi:10.1016/j.neuron.2017.07.011. 28757305 PMC5576360

[B38] Hacker CD, Laumann TO, Szrama NP, Baldassarre A, Snyder AZ, Leuthardt EC, Corbetta M. Resting state network estimation in individual subjects. Neuroimage 82: 616–633, 2013. doi:10.1016/j.neuroimage.2013.05.108. 23735260 PMC3909699

[B39] Hampson M, Peterson BS, Skudlarski P, Gatenby JC, Gore JC. Detection of functional connectivity using temporal correlations in MR images. Hum Brain Mapp 15: 247–262, 2002. doi:10.1002/hbm.10022. 11835612 PMC6872035

[B40] Hein G, Knight RT. Superior temporal sulcus—It’s my area: or is it? J Cogn Neurosci 20: 2125–2136, 2008. doi:10.1162/jocn.2008.20148. 18457502

[B41] Hesselmann V, Sorger B, Lasek K, Guntinas-Lichius O, Krug B, Sturm V, Goebel R, Lackner K. Discriminating the cortical representation sites of tongue and lip movement by functional MRI. Brain Topogr 16: 159–167, 2004. doi:10.1023/B:BRAT.0000019184.63249.e8. 15162913

[B42] Hickok G, Poeppel D. The cortical organization of speech processing. Nat Rev Neurosci 8: 393–402, 2007. doi:10.1038/nrn2113. 17431404

[B43] Jakobsen E, Liem F, Klados MA, Bayrak Ş, Petrides M, Margulies DS. Automated individual-level parcellation of Broca’s region based on functional connectivity. Neuroimage 170: 41–53, 2018. doi:10.1016/j.neuroimage.2016.09.069. 27693796

[B44] Ji JL, Spronk M, Kulkarni K, Repovš G, Anticevic A, Cole MW. Mapping the human brain’s cortical-subcortical functional network organization. Neuroimage 185: 35–57, 2019. doi:10.1016/j.neuroimage.2018.10.006. 30291974 PMC6289683

[B45] Kong R, Li J, Orban C, Sabuncu MR, Liu H, Schaefer A, Sun N, Zuo XN, Holmes AJ, Eickhoff SB, Yeo BTT. Spatial topography of individual-specific cortical networks predicts human cognition, personality, and emotion. Cereb Cortex 29: 2533–2551, 2019. doi:10.1093/cercor/bhy123. 29878084 PMC6519695

[B46] Krubitzer L. The magnificent compromise: cortical field evolution in mammals. Neuron 56: 201–208, 2007. doi:10.1016/j.neuron.2007.10.002. 17964240

[B47] Kwong KK, Belliveau JW, Chesler DA, Goldberg IE, Weisskoff RM, Poncelet BP, Kennedy DN, Hoppel BE, Cohen MS, Turner R. Dynamic magnetic resonance imaging of human brain activity during primary sensory stimulation. Proc Natl Acad Sci USA 89: 5675–5679, 1992. doi:10.1073/pnas.89.12.5675. 1608978 PMC49355

[B48] Laumann TO, Gordon EM, Adeyemo B, Snyder AZ, Joo SJ, Chen MY, Gilmore AW, McDermott KB, Nelson SM, Dosenbach NU, Schlaggar BL, Mumford JA, Poldrack RA, Petersen SE. Functional system and areal organization of a highly sampled individual human brain. Neuron 87: 657–670, 2015. doi:10.1016/j.neuron.2015.06.037. 26212711 PMC4642864

[B49] Lee MH, Hacker CD, Snyder AZ, Corbetta M, Zhang D, Leuthardt EC, Shimony JS. Clustering of resting state networks. PLoS One 7: e40370, 2012. doi:10.1371/journal.pone.0040370. 22792291 PMC3392237

[B50] Mahowald K, Fedorenko E. Reliable individual-level neural markers of high-level language processing: a necessary precursor for relating neural variability to behavioral and genetic variability. Neuroimage 139: 74–93, 2016. doi:10.1016/j.neuroimage.2016.05.073. 27261158

[B51] Marcus DS, Harwell J, Olsen T, Hodge M, Glasser MF, Prior F, Jenkinson M, Laumann T, Curtiss SW, Van Essen DC. Informatics and data mining tools and strategies for the human connectome project. Front Neuroinform 5: 4, 2011. doi:10.3389/fninf.2011.00004. 21743807 PMC3127103

[B52] Margulies DS, Ghosh SS, Goulas A, Falkiewicz M, Huntenburg JM, Langs G, Bezgin G, Eickhoff SB, Castellanos FX, Petrides M, Jefferies E, Smallwood J. Situating the default-mode network along a principal gradient of macroscale cortical organization. Proc Natl Acad Sci USA 113: 12574–12579, 2016. doi:10.1073/pnas.1608282113. 27791099 PMC5098630

[B53] Margulies DS, Vincent JL, Kelly C, Lohmann G, Uddin LQ, Biswal BB, Villringer A, Castellanos FX, Milham MP, Petrides M. Precuneus shares intrinsic functional architecture in humans and monkeys. Proc Natl Acad Sci USA 106: 20069–20074, 2009. doi:10.1073/pnas.0905314106. 19903877 PMC2775700

[B54] Mennes M, Jenkinson M, Valabregue R, Buitelaar JK, Beckmann C, Smith S. Optimizing full-brain coverage in human brain MRI through population distributions of brain size. Neuroimage 98: 513–520, 2014. doi:10.1016/j.neuroimage.2014.04.030. 24747737

[B55] Mesulam MM. A cortical network for directed attention and unilateral neglect. Ann Neurol 10: 309–325, 1981. doi:10.1002/ana.410100402. 7032417

[B56] Mesulam MM. Large-scale neurocognitive networks and distributed processing for attention, language, and memory. Ann Neurol 28: 597–613, 1990. doi:10.1002/ana.410280502. 2260847

[B57] Mesulam MM, Wieneke C, Hurley R, Rademaker A, Thompson CK, Weintraub S, Rogalski EJ. Words and objects at the tip of the left temporal lobe in primary progressive aphasia. Brain 136: 601–618, 2013. doi:10.1093/brain/aws336. 23361063 PMC3572925

[B58] Mineroff Z, Blank IA, Mahowald K, Fedorenko E. A robust dissociation among the language, multiple demand, and default mode networks: Evidence from inter-region correlations in effect size. Neuropsychologia 119: 501–511, 2018. doi:10.1016/j.neuropsychologia.2018.09.011. 30243926 PMC6191329

[B59] Mirman D, Chen Q, Zhang Y, Wang Z, Faseyitan OK, Coslett HB, Schwartz MF. Neural organization of spoken language revealed by lesion-symptom mapping. Nat Commun 6: 6762, 2015. doi:10.1038/ncomms7762. 25879574 PMC4400840

[B60] Mueller S, Wang D, Fox MD, Yeo BTT, Sepulcre J, Sabuncu MR, Shafee R, Lu J, Liu H. Individual variability in functional connectivity architecture of the human brain. Neuron 77: 586–595, 2013. doi:10.1016/j.neuron.2012.12.028. 23395382 PMC3746075

[B61] Ogawa S, Tank DW, Menon R, Ellermann JM, Kim SG, Merkle H, Ugurbil K. Intrinsic signal changes accompanying sensory stimulation: functional brain mapping with magnetic resonance imaging. Proc Natl Acad Sci USA 89: 5951–5955, 1992. doi:10.1073/pnas.89.13.5951. 1631079 PMC402116

[B62] Petersen SE, Fox PT, Posner MI, Mintun M, Raichle ME. Positron emission tomographic studies of the cortical anatomy of single-word processing. Nature 331: 585–589, 1988. doi:10.1038/331585a0 . 3277066

[B63] Petrides M, Cadoret G, Mackey S. Orofacial somatomotor responses in the macaque monkey homologue of Broca’s area. Nature 435: 1235–1238, 2005. doi:10.1038/nature03628. 15988526

[B64] Poldrack RA, Laumann TO, Koyejo O, Gregory B, Hover A, Chen MY, Gorgolewski KJ, Luci J, Joo SJ, Boyd RL, Hunicke-Smith S, Simpson ZB, Caven T, Sochat V, Shine JM, Gordon E, Snyder AZ, Adeyemo B, Petersen SE, Glahn DC, Reese Mckay D, Curran JE, Göring HH, Carless MA, Blangero J, Dougherty R, Leemans A, Handwerker DA, Frick L, Marcotte EM, Mumford JA. Long-term neural and physiological phenotyping of a single human. Nat Commun 6: 8885, 2015. doi:10.1038/ncomms9885. 26648521 PMC4682164

[B65] Poldrack RA, Wagner AD, Prull MW, Desmond JE, Glover GH, Gabrieli JD. Functional specialization for semantic and phonological processing in the left inferior prefrontal cortex. Neuroimage 10: 15–35, 1999. doi:10.1006/nimg.1999.0441. 10385578

[B66] Power JD, Cohen AL, Nelson SM, Wig GS, Barnes KA, Church JA, Vogel AC, Laumann TO, Miezin FM, Schlaggar BL, Petersen SE. Functional network organization of the human brain. Neuron 72: 665–678, 2011. doi:10.1016/j.neuron.2011.09.006. 22099467 PMC3222858

[B67] Scott TL, Gallée J, Fedorenko E. A new fun and robust version of an fMRI localizer for the frontotemporal language system. Cogn Neurosci 8: 167–176, 2017. doi:10.1080/17588928.2016.1201466. 27386919

[B68] Seeley WW, Menon V, Schatzberg AF, Keller J, Glover GH, Kenna H, Reiss AL, Greicius MD. Dissociable intrinsic connectivity networks for salience processing and executive control. J Neurosci 27: 2349–2356, 2007. doi:10.1523/JNEUROSCI.5587-06.2007. 17329432 PMC2680293

[B69] Setsompop K, Gagoski BA, Polimeni JR, Witzel T, Wedeen VJ, Wald LL. Blipped-controlled aliasing in parallel imaging for simultaneous multislice echo planar imaging with reduced g-factor penalty. Magn Reson Med 67: 1210–1224, 2012. doi:10.1002/mrm.23097. 21858868 PMC3323676

[B70] Smith SM, Fox PT, Miller KL, Glahn DC, Fox PM, Mackay CE, Filippini N, Watkins KE, Toro R, Laird AR, Beckmann CF. Correspondence of the brain’s functional architecture during activation and rest. Proc Natl Acad Sci USA 106: 13040–13045, 2009. doi:10.1073/pnas.0905267106. 19620724 PMC2722273

[B71] Smith SM, Jenkinson M, Woolrich MW, Beckmann CF, Behrens TEJ, Johansen-Berg H, Bannister PR, De Luca M, Drobnjak I, Flitney DE, Niazy RK, Saunders J, Vickers J, Zhang Y, De Stefano N, Brady JM, Matthews PM. Advances in functional and structural MR image analysis and implementation as FSL. Neuroimage 23, Suppl 1: S208–S219, 2004. doi:10.1016/j.neuroimage.2004.07.051. 15501092

[B72] Tavor I, Parker Jones O, Mars RB, Smith SM, Behrens TE, Jbabdi S. Task-free MRI predicts individual differences in brain activity during task performance. Science 352: 216–220, 2016. doi:10.1126/science.aad8127. 27124457 PMC6309730

[B73] Thompson-Schill SL, D’Esposito M, Aguirre GK, Farah MJ. Role of left inferior prefrontal cortex in retrieval of semantic knowledge: a reevaluation. Proc Natl Acad Sci USA 94: 14792–14797, 1997. doi:10.1073/pnas.94.26.14792. 9405692 PMC25116

[B74] van der Kouwe AJ, Benner T, Fischl B, Schmitt F, Salat DH, Harder M, Sorensen AG, Dale AM. On-line automatic slice positioning for brain MR imaging. Neuroimage 27: 222–230, 2005. doi:10.1016/j.neuroimage.2005.03.035. 15886023

[B75] van der Kouwe AJ, Benner T, Salat DH, Fischl B. Brain morphometry with multiecho MPRAGE. Neuroimage 40: 559–569, 2008. doi:10.1016/j.neuroimage.2007.12.025. 18242102 PMC2408694

[B76] Van Essen DC, Smith SM, Barch DM, Behrens TE, Yacoub E, Ugurbil K; WU-Minn HCP Consortium. The WU-Minn Human Connectome Project: an overview. Neuroimage 80: 62–79, 2013. doi:10.1016/j.neuroimage.2013.05.041. 23684880 PMC3724347

[B77] Vincent JL, Kahn I, Snyder AZ, Raichle ME, Buckner RL. Evidence for a frontoparietal control system revealed by intrinsic functional connectivity. J Neurophysiol 100: 3328–3342, 2008. doi:10.1152/jn.90355.2008. 18799601 PMC2604839

[B78] Weiskopf N, Hutton C, Josephs O, Deichmann R. Optimal EPI parameters for reduction of susceptibility-induced BOLD sensitivity losses: a whole-brain analysis at 3 T and 1.5 T. Neuroimage 33: 493–504, 2006. doi:10.1016/j.neuroimage.2006.07.029. 16959495

[B79] Wise R, Chollet F, Hadar U, Friston K, Hoffner E, Frackowiak R. Distribution of cortical neural networks involved in word comprehension and word retrieval. Brain 114: 1803–1817, 1991. doi:10.1093/brain/114.4.1803. 1884179

[B80] Woolrich MW, Ripley BD, Brady M, Smith SM. Temporal autocorrelation in univariate linear modeling of FMRI data. Neuroimage 14: 1370–1386, 2001. doi:10.1006/nimg.2001.0931. 11707093

[B81] Xu J, Moeller S, Strupp J, Auerbach E, Feinberg DA, Ugurbil K, Yacoub E. Highly accelerated whole brain imaging using aligned-blipped-controlled-aliasing multiband EPI. Proc Int Soc Magn Reson Med 20: 2306, 2012.

[B82] Yeo BTT, Krienen FM, Sepulcre J, Sabuncu MR, Lashkari D, Hollinshead M, Roffman JL, Smoller JW, Zöllei L, Polimeni JR, Fischl B, Liu H, Buckner RL. The organization of the human cerebral cortex estimated by intrinsic functional connectivity. J Neurophysiol 106: 1125–1165, 2011. doi:10.1152/jn.00338.2011. 21653723 PMC3174820

